# Exploring the molecular content of CHO exosomes during bioprocessing

**DOI:** 10.1007/s00253-021-11309-8

**Published:** 2021-05-03

**Authors:** Christoph Keysberg, Oliver Hertel, Louise Schelletter, Tobias Busche, Chiara Sochart, Jörn Kalinowski, Raimund Hoffrogge, Kerstin Otte, Thomas Noll

**Affiliations:** 1 Bielefeld University, Bielefeld, Germany; 2 University of Applied Sciences Biberach, Biberach, Germany; 3 Center for Biotechnology (CeBiTec), Bielefeld University, Bielefeld, Germany

**Keywords:** Biotechnology, CHO, Exosomes, Proteomics, miRNA, piRNA

## Abstract

**Abstract:**

In biopharmaceutical production, Chinese hamster ovary (CHO) cells derived from *Cricetulus griseus* remain the most commonly used host cell for recombinant protein production, especially antibodies. Over the last decade, in-depth multi-omics characterization of these CHO cells provided data for extensive cell line engineering and corresponding increases in productivity. However, exosomes, extracellular vesicles containing proteins and nucleic acids, are barely researched at all in CHO cells. Exosomes have been proven to be a ubiquitous mediator of intercellular communication and are proposed as new biopharmaceutical format for drug delivery, indicator reflecting host cell condition and anti-apoptotic factor in spent media. Here we provide a brief overview of different separation techniques and subsequently perform a proteome and regulatory, non-coding RNA analysis of exosomes, derived from lab-scale bioreactor cultivations of a CHO-K1 cell line, to lay out reference data for further research in the field. Applying bottom-up orbitrap shotgun proteomics and next-generation small RNA sequencing, we detected 1395 proteins, 144 micro RNA (miRNA), and 914 PIWI-interacting RNA (piRNA) species differentially across the phases of a batch cultivation process. The exosomal proteome and RNA data are compared with other extracellular fractions and cell lysate, yielding several significantly exosome-enriched species.

**Figure:**
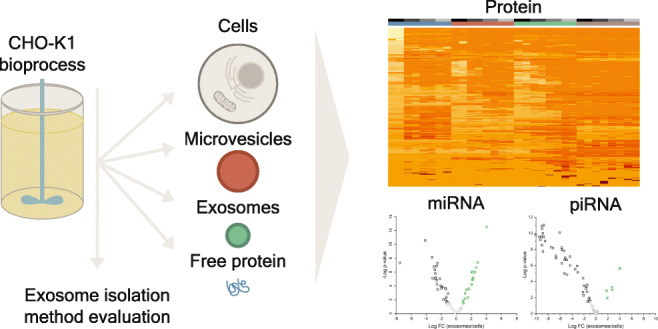
Graphical Abstract

**Key points:**

• *First-time comprehensive protein and miRNA characterization of CHO exosomes.*

• *Isolation protocol and time point of bioprocess strongly affect quality of extracellular vesicles.*

• *CHO-derived exosomes also contain numerous piRNA species of yet unknown function.*

**Supplementary Information:**

The online version contains supplementary material available at 10.1007/s00253-021-11309-8.

## Introduction

Chinese hamster ovary (CHO) cells are widely used host cells for recombinant protein production and currently the most commonly utilized mammalian organism in large scale biopharmaceutical production (Fischer et al. 2015). Particularly, therapeutics and monoclonal antibodies (mAbs) are predominantly expressed in CHO cells. Status as of 2018, 57 out of 68 commercially available mAbs, i.e., 87%, were produced in CHO cells, equating to a marked value of 107 billion dollars (Walsh 2018). CHO cells can produce large quantities of human-like post-translational modified proteins that are suitable for application as therapeutics, with significantly better growth rates than human cell lines. Compared to non-mammalian expression-systems, however, their cultivation is challenging, which is why their usage is only economical for the production of more complex therapeutics that require human-like post-translational modifications for efficacy or tolerance (Fischer et al. 2015).

Exosomes are 30–150 nm small vesicles, that derive from the endosomal network and can therefore be distinguished from plasma membrane-shed microvesicles (100–1000 nm in diameter) and apoptotic vesicles (50–5000 nm), which are secluded over the course of programmed cell death. Since the discovery of exosomes as a new extracellular vesicle (EV) subspecies by Johnstone et al. (1987) it turned out exosomes are not only vehicles of cellular waste disposal, as initially assumed, but also a conserved mechanism of cellular communication (Colombo et al. 2014). While research in the field increased exponentially in the last decade, there is barely any research on CHO cell derived exosomes yet.

Recently, exosomes were intensively studied in immunological and oncological contexts. The finding of ribonucleic acid (RNA) cargo in exosomes (Valadi et al. 2007), including fully functional messenger RNA (mRNA), uncovered a previously unknown but surprisingly common process of horizontal RNA transfer. The exosomal RNA is not randomly applied but specifically sorted, processed and wrapped into the vesicles (Ramachandran and Palanisamy 2012), sometimes co-packaged with adenine phosphoribosyltransferases, e.g., miRNA already bound to RNA-induces silencing complex (RISC) (Turchinovich et al. 2011). MicroRNAs (miRNAs) have been reported to mediate intercellular communication on a transcriptional level in context of angiogenesis, hematopoiesis and tumorigenesis (Waldenström and Ronquist 2014). Moreover, non-coding RNA (ncRNA) species were found to be specifically loaded into tumor-derived exosomes (Kogure et al. 2013) causing a pro-metastatic development on artificial application (Conigliaro et al. 2015), thus marking the importance of exosome-derived RNAs in (patho-)physiology in vivo.

Because of their systemic mode of action, miRNAs are potentially effective regulators of exosome-mediated communication that could be used for biotechnological approaches using exosomes as drug-delivery vehicles (El-Andaloussi et al. 2012). In regard of cell line engineering, there have been several miRNA screenings investigating targets and respective phenotypes in CHO cells (Müller et al. 2008; Hackl et al. 2011; Fischer et al. 2014; Bischoff et al. 2015). However, except for miRNA and the more specific small-interfering RNA (siRNA), a recent study predicted a third group of ncRNA in CHO cells: PIWI-interacting RNA (piRNA) (Gerstl et al. 2013). These are 24–31 nt small ncRNAs, discovered in 2006 (Aravin et al. 2006; Girard et al. 2006; Lau et al. 2006) that interact with the argonaute protein-related PIWI-proteins (Iwasaki et al. 2015). Originally, piRNA was thought to be a germline-specific species involved in the degradation of complementary transposon transcripts (Siomi et al. 2011). However, later works demonstrated that piRNAs also play an important role in epigenetic regulation, facilitating histone modification and deoxyribonucleic acid (DNA) methylation in somatic cells (Peng and Lin 2013; Ross et al. 2014). Moreover, it was shown that piRNAs also act in a regulatory manner that can sometimes be very similar to miRNA (Peng et al. 2016). Although piRNA functions are not fully understood yet and there are even fewer studies focusing on piRNA in exosomes (Yang et al. 2018; Jain et al. 2019; Pippadpally and Venkatesh 2020), they pose an interesting species for future cell line engineering or even therapeutic approaches.

The only studies conducted yet on the matter of CHO exosomes claimed an increased growth (Takagi et al. 2020) and attenuated apoptosis mediated by exosomes (Han and Rhee 2018). Others suggest exosomes as potential parameter for industrial bioprocess monitoring (Zavec et al. 2016). Moreover, exosomes are considered potential drug delivery vehicle, due to their specific tropism, proposedly weak immunogenicity and the ability to cross the blood-brain-barrier (Liu and Su 2019). Therefore, tailored exosomes packed up with small molecules (Lamichhane and Jay 2018), miRNA (Zeh et al. 2019) or Fab fragments attached to their surface (Longatti et al. 2018) have been studied.

A major hurdle in exosome research is the lack of a “gold-standard” isolation method. According to a worldwide study in 2016, most labs (81%) use ultracentrifugation-based methods (Gardiner et al. 2016). Ultracentrifugation protocols seem to be widely applicable and robust, but suffer from long preparation time, small yield, limited scalability and irreversible aggregation of particles. Filtration or precipitation steps can sometimes substitute centrifugation and speed up the process and provide more scalability (Heinemann and Vykoukal 2017). Precipitation can be achieved, e.g., with salts like NaOAc (Brownlee et al. 2014), polyethylene glycol (PEG) (Rider et al. 2016) or commercially available kits. However, the purity of the recovered material was often shown to be lacking (Patel et al. 2019). Alternatively, size exclusion chromatography poses a quick and scalable option for exosome separation. Its biggest downside is the inherent dilution and lack of particle concentration in the acquired fractions, calling for further steps (Lobb and Möller 2017). The presumably best method in terms of exosome purity is immunocapture. This is also the most expensive approach though and requires detailed knowledge about the surface structure of the aimed vesicle population. In many cases researchers may lack this information and it was recently shown that even supposedly universal exosome markers such as CD9, CD63 and CD81 are differentially expressed in several exosome and microvesicle subtypes (Jeppesen et al. 2019), probably also differing between host cell types and species.

This work aims to outline possible separation techniques followed by a differential proteomic and transcriptomic characterization of CHO EVs, especially exosomes, over the course of a bioreactor batch cultivation. Therefore, a protocol yielding small EVs with a strong exosomal marker enrichment is determined to subsequently compare the exosome composition with those of larger microvesicles, host cell protein, and cell lysate from the same batch process at different time points.

## Materials and methods

### Cell culture

Chinese hamster ovary (CHO) K1 suspension cells (strain ATCC 61-CCL) were cultivated in shake flasks at 185 rpm, 37 °C, 5% CO_2_, and 80% humidity in a Mytron cell culture incubator (Memmert; Schwabach, Germany) or in 2 l B-DCU bioreactors (Sartorius; Göttingen, Germany). To determine viable cell density and viability, they were counted with a Cedex™ automated cell counter device (Roche; Basel, Switzerland). Cultivation took place in serum-free TCX6D medium (Xell; Bielefeld, Germany), supplemented with 8 mM glutamine.

### Differential ultracentrifugation

Exosome separation via ultracentrifugation was performed with modifications after Théry et al. (2006). Culture broth was centrifuged 5 min at 500*g* and 30 min at 2000*g* thereafter, transferring the respective supernatant. After filtering the supernatant with 0.22 μm minisart filters (Sartorius; Göttingen, Germany), it was concentrated up to 50-fold in Vivaspin 20, 100 kDa cut-off centrifugation tubes (Sartorius; Göttingen, Germany). The concentrate was inserted for a two-step ultracentrifugation at 100,000*g* for 70 min, washing the exosome-pellet with 1 ml phosphate buffered saline (PBS), containing 1 mM phenylmethylsulfonyl fluoride (PMSF) as protease inhibitor. The final exosome pellet was taken up in 100 μl PBS with 1 mM PMSF. For the growth phase experiment, microvesicles were sedimented at 20,000*g* for 30 min prior to 0.22 μm filtration and washed once in PBS. The pellet was taken up in 100 μl PBS with 1 mM PMSF. Host cell protein was concentrated from the 100 kDa cut-off flow through in 3 kDa cut-off centrifugation tubes (Sartorius; Göttingen, Germany) and precipitated twice with 90% acetone.

### Lower speed centrifugation

Cells were sedimented at 300*g* for 10 min. Debris and larger microparticles were subsequently depleted centrifuging the supernatant for 30 min at 2000*g* and then for 45 min at 10,000*g*. Transferring the supernatant, exosomes were then sedimented for 3 h at 20,000*g*, washed in 3 ml PBS with 1 mM PMSF, and again centrifuged at 20,000*g* for 3 h. The resulting pellet was resuspended in 100 μL PBS with 1 mM PMSF.

### ExoQuick-TC™

The ExoQuick-TC™ exosome isolation kit (System Biosciences; Palo Alto CA, USA) was used according to the manufacturer’s instructions on concentrated culture supernatant. Cells and bigger particles were removed by centrifugation at 3000*g* for 15 min. The supernatant was then added with 1/6 volumes of ExoQuick-TC™ precipitation reagent. The reagent was mixed with the supernatant and stored at 4 °C overnight. The next day, the sample was centrifuged twice at 1500*g* for 30 min and 5 min, respectively, to take off the supernatant. The pellet was resuspended in 100 μl PBS, supplemented with 1 mM PMSF.

### PEG precipitation

Precipitation with polyethylene glycol was carried out as noted by Rider et al*.* (2016). Culture broth was centrifuged 5 min at 500*g* and 30 min at 2000*g*. The precipitation mixture (16% PEG6000 (Merck; Darmstadt, Germany), 1 mM NaCl), was added 1:1 (v/v) to the supernatant, mixed and stored at 4 °C overnight. Precipitated components were sedimented by centrifugation at 4000*g* for 75 min. The supernatant was thoroughly removed, and the pellet was taken off in PBS and transferred into a 13 ml polypropylene tube (Beckman Coulter; Brea CA, USA) before being centrifuged at 100,000*g* for 70 min using a TH-641 rotor (Thermo Fisher Scientific; Waltham MA, USA) in an Ultra-Pro 80 centrifuge (Thermo Fisher Scientific; Waltham MA, USA). The exosome-pellet was resuspended in 100 μl PBS with 1 mM PMSF.

### Sodium acetate precipitation

NaOAc precipitation complied with the protocol reported by Brownlee et al*.* (2014). Centrifugation of the supernatant took place at 500*g* for 30 min and another 30 min at 10,000*g*, transferring the supernatant. 0.1 volumes of 1 M NaOAc solution (pH 4.75) were added, mixed and stored 60 min on ice, followed by 5 min at 37 °C. The precipitate was then centrifuged at 5000*g* for 10 min, resuspended in 0.1 M NaOAc and again centrifuged for 10 min at 5000*g* to wash it. The resulting pellet was taken off in 100 μl PBS with 1 mM PMSF.

### Protein extraction and quantification

Cell samples for lysis and protein extraction were collected parallelly to exosome separation by taking off 1E7 cells, washing them twice for 5 min at 200*g* in cold PBS. The cell and EV samples were stored at −80 °C until thawing. Cell pellets were resuspended in 200 μl ice-cold lysis buffer (50 mM Tris-HCl pH 7.2, 150 mM NaCl, 2 mM ethylenediaminetetraacetic acid, 1 mM PMSF, 0.1% sodium dodecyl sulfate, 1% nonyl phenoxypolyethoxylethanol) by vortexing. EV suspensions were added with 1 volume of 2 X lysis buffer instead. After 5 min on ice, the sample was treated 5 min with ultrasonic sound and stored on ice for 30 min, before being centrifuged at 16,200*g* for 30 min to remove debris. Determination of total protein concentration was conducted in duplicates via Bicinchoninic Acid (BCA) Assay Protein Quantitation Kit (Thermo Fisher Scientific; Waltham MA, USA) according to the manufacturer’s instructions measuring the extinction at 570 nm with a PowerWave HT microplate reader (BioTek; Winooski VT, USA).

### SDS-PAGE and western blot

Sodium dodecyl sulfate-polyacrylamide gel electrophoresis (SDS-PAGE) was done using a 14% running and a 4% stocking gel. A total of 20 μg of sample protein was mixed with Laemmli loading buffer (2% sodium dodecyl sulfate, 62.5 mM Tris-HCl pH 6.8, 10% glycerol, 0.01% bromphenol blue), reducing agent (50 mM dithiothreitol) and heat denaturated. For western blot, proteins were transferred onto a polyvinylidene fluoride membrane (BioRad; Hercules CA, USA) at 35 V for 60 min. Primary antibodies anti-Tsg101 (ABIN2780037, antibodies-online) and anti-CD81 (LS-C108453, LSBio) were applied 1000-fold diluted in 5% bovine serum albumin. Detection took place with Cy3-conjugated anti-rabbit secondary antibodies in an Ettan™ DIGE Imager (GE Healthcare; Chicago IL, USA).

### Tryptic digest and mass spectrometry

Prior to digestion, samples were desalted with 7 K cut-off Zeba™ Spin Desalting Columns (Thermo Fisher Scientific; Waltham MA, USA). Samples were then reduced with 7 mM dithiothreitol 30 min at 60 °C and alkylated with 20 mM iodacetamide for 30 min in the dark at room temperature (RT; 20–22 °C). Reaction stop was induced by the incubation of 14 mM dithiothreitol for 45 min. Twenty microgram protein was digested overnight with 10 ng Trypsin Gold (Promega; Madison WI, USA) per 1.5 μg protein at pH 8.5. Samples were then purified with Sep-Pak® C18 Cartridges (Waters; Milford MA, USA). The peptide extract was dried out in a vacuum concentrator and resuspended in 2.5% acetonitrile/0.1% trifluoroacetic acid in LiChrosolv® (Merck; Darmstadt, Germany). Nanoscale liquid chromatography was conducted with 2 μg peptides on a PepMap™ *100* C18 trap column (Thermo Scientific; Waltham MA, USA) and a 25 cm PepMap™ C18 separation column (Thermo Scientific; Waltham MA, USA) with a flow of 300 nl min^−1^. For elution, an increasing 60 min acetonitrile gradient peaking at 76% acetonitrile (v/v) was applied. Mass spectrometry measurements (MS) were performed with a Q Exactive Plus Orbitrap MS (Thermo Scientific; Waltham MA, USA) in positive mode, a mass range between m/z 350 to 1600 and tandem MS detection in data-dependent acquisition mode (top 10 method). Spectra were matched against TrEMBL database for *Cricetulus griseus* and *Mus musculus* via MaxQuant 1.6.10.43 (Max Planck Institute of Biochemistry; Munich, Germany). Peptides were filtered for max. two missed cleavages, lengths of 6–150 amino acids, 10 ppm precursor and 0.6 Da fragment mass tolerance with max. three equal modifications. Oxidation (M, +15.995 Da), acetylation (N-terminus, +42.011 Da), Met-loss (N-terminus, −131.040 Da), and Met-loss + acetylation (N-terminus, −89.030 Da) were specified as dynamic modifications and carbamidomethylation (C, +57.021 Da) was set as static modification. The gene ontology enrichment analysis was performed with DAVID (Huang et al. 2009a; Huang et al. 2009b). Other statistical tests were calculated via Perseus 1.6.10.45 (Tyanova and Cox 2018), with log2-transformed label-free quantification intensities. Two-sided Student’s *t*-tests were done with a false discovery rate-threshold of 0.05 and S0 of 0.1. For principal component analysis, only proteins with > 70% valid values were used.

### Transmission electron microscopy

Transmission electron microscopy was conducted using an EM 109 electron microscope (Carl Zeiss; Oberkochen, Germany). EV suspensions in PBS were loaded onto discharge-treated 3.05 mm copper grids. After 2 min of incubation, the sample was fixed and stained with 1% uranyl acetate solution three times. Pictures were taken in 50 kV-mode at 50,000-fold magnification and 2 s exposure time.

### RNA extraction and library construction

For total RNA extraction, cell pellets were resuspended in 600 μl TRI Reagent (Zymo Research; Irvine CA, USA) and EV pellets were resuspended in 100 μl PBS and 300 μl TRI Reagent was added. The lysed samples were mixed with 200 μl (EV 100 μl) chloroform and were centrifuged at 12,000*g*, 4 °C for 15 min. The aqueous phase was taken of and extracted again with 500 μl (EV 300 μl) TRI Reagent and 200 μl (EV 100 μl) chloroform. RNA was precipitated from the aqueous phase by incubation with the same volume isopropanol and centrifuged at 12,000*g*, 4 °C for 10 min. The RNA pellet was washed with 1 mL (EV 300 μl) 75% ethanol twice. After drying, the pellet was resuspended in 50 μl (EV 10 μl) RNase-free water.

### Small RNA sequencing and data analysis

One hundred nanogram exosomal RNA were used to prepare small strand-specific RNA-Seq libraries with the Small RNA-Seq Library Prep Kit for Illumina (Lexogen; Vienna, Austria) according to the manufacturer’s instructions. Size selection of small RNA-Seq libraries and adapter removal was performed with BluePippin (Sage Science; Beverly MA, USA) using a 3% agarose standard DNA size selection cassette selecting fragments ranging from 137 to 162 nt, which includes inserts from 15 to 40 nt. Prior to sequencing, the indexed libraries were quantified using the Qubit (Thermo Fisher Scientific; Waltham MA, USA) and the Agilent Bioanalyzer 2100 (Agilent; Santa Clara CA, USA). The libraries were pooled and sequenced in paired-end mode on an Illumina MiSeq system using a read length of 36 nt. Only the forward reads were used for analysis. Quality control of raw reads was conducted by FastQC 0.11.8 (Andrews 2010). Lexogen sequencing adapters were removed by Cutadapt 2.9 (Martin 2011) and reads shorter than 15 bases were discarded. The remaining reads were aligned to the *C. griseus* RefSeq assembly GCF_003668045.1 by Bowtie 1.2.3 (Langmead et al. 2009) in -v1 alignment and best alignment reporting mode. Mapped reads were filtered with samtools 1.10-31 (Li et al. 2009) and used as high-quality reads for transcriptome mapping. To identify different RNA species without affecting the mapping by arranging the order of mapping to different references, miRNA sequences were removed from the *C. griseus* RefSeq transcriptome GCF_003668045.1. Then, piRNAs from piRNAbank 1.7.6 (Sai lakshmi and Agrawal 2008) and hairpin structures from miRbase release 22.1 (Kozomara and Griffiths-Jones 2011) were concatenated with the RefSeq transcriptome. High-quality reads were mapped on this custom transcriptome by Bowtie1 in -l 8 -n 1 alignment and best alignment reporting mode. Reads were counted by samtools idxstats (Li et al. 2009) and features with less than three reads were filtered out. The raw counts were then annotated with their respective RNA species. Quantification of miRNAs and piRNAs was done by edgeR 3.28.1 (Robinson et al. 2009). Raw counts were merged and two data frames, exosomes vs cell lysate and high viability (logarithmic and stationary growth phase) vs low viability (death phase), were defined. Counts were filtered by expression with default values and normalized with trimmed mean of M values (Robinson and Oshlack 2010).

## Results

### Evaluation of exosome separation methods

In order to identify an appropriate separation technique for CHO exosomes, different methods were compared including a classic ultracentrifugation, a prolonged centrifugation at sub-ultracentrifugation acceleration (3 h at 20,000*g*), the commercial ExoQuick-TC™ kit, as well as PEG- and NaOAc-based precipitation protocols. To ensure comparability of methods, similar input volumes of 200 ml harvested CHO-K1 suspension culture (pooled, 7.4E6 cells per ml and 98% viability) each from logarithmic growth phase were applied to test for exosome separation efficacy of different protocols. The five protocols were conducted as proposed by the manufacturer or as described in the methods, respectively. Two other immunoaffinity-based commercial kits against murine and human exosome markers were also evaluated. However, these did not yield any detectable amount of protein or particles for up to 200 ml input volume and are therefore not discussed in the following.

**Fig. 1:**
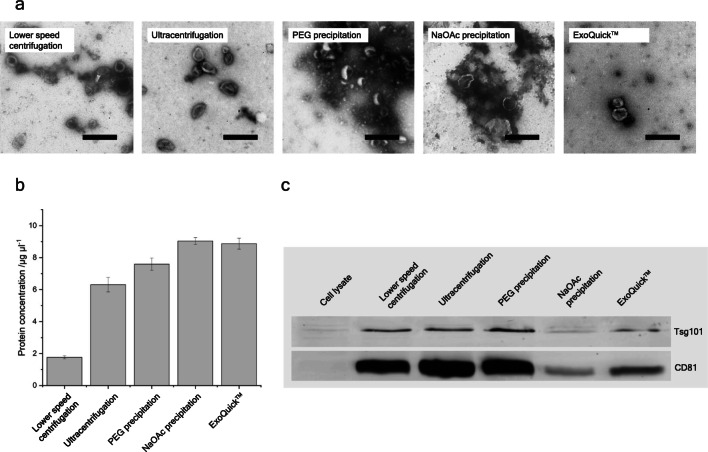
**a** Transmission electron microscopy of exosome samples. Compared samples are derived by a classic ultracentrifugation, a prolonged centrifugation at sub-ultracentrifugation acceleration (3 h at 20,000*g*), the commercially available ExoQuick^TM^ Kit, a sodium acetate precipitation (NaOAc), and a polyethylene glycol precipitation (PEG). Scale bar: 400 nm. **b** Bar chart comparing protein concentration yields from the abovementioned protocols. Eventually all samples were taken up in 100 μl PBS with PMSF. **c** Western blot with samples from different purification methods and CHO cell lysate against the exosomal proteins Tsg101 and CD81. Same protein amounts (20 μg) were loaded for each lane

To verify the presence of vesicles, transmission electron microscopy was performed after exosome separation (cf. Fig. 1a). The lower speed centrifugation sample rendered exosomes with typical “cup-shaped” appearance, resulting from the fixation process of EVs. While some particles were > 300 nm in diameter and thus expected to be microvesicles, others with diameters ≤ 100 nm are most certainly exosomes. In the sample derived from the classical ultracentrifugation protocol particles showed a similar appearance but displaying a lower polydispersity. Most of them were around the commonly acclaimed size range of exosomes (30–150 nm in diameter), but some structures appeared slightly larger. Some sample inhomogeneity is to be expected, especially considering the in part overlapping size range of exosomes and microvesicles. The PEG-precipitated sample was clouded with a smear and contained vesicle structures with diameters between approx. 50 and 200 nm, suggesting a mixed EV composition that was mostly exosome-like in size, but not pure in the strict sense. While the NaOAc-precipitated sample also displayed vesicle structures in electron microscopy, those were largely clouded by a smear. The vesicles themselves ranged from 30 up to 300 nm in size. In the ExoQuick-TC™ sample, some vesicles of approx. 100–300 nm in diameter were visible, which fits with the manufacturer’s declarations (System Biosciences 2016) but is not the commonly accepted size range of exosomes (Théry et al. 2018), indicating a similarly mixed population as in the NaOAc-precipitated sample.

The protein determination of the exosome preparations via BCA showed that the recovered protein amounts differed greatly of up to approx. 5-fold between the different methods (cf. Fig. 1b). In general, the precipitation-based methods yielded the highest protein concentrations with 9.04 (NaOAc), 8.88 (ExoQuick-TC™), and 7.59 μg μl^−1^ (PEG). While the ultracentrifugation sample contained a medium protein amount of 6.31 μg μl^−1^, the lower speed centrifugation protocol yielded the lowest protein amounts of only 1.77 μg μl^−1^.

To verify the endosomal origin of the purified exosomes, a western blot against the exosome markers CD81 and Tsg101 was performed and showed a clear enrichment of both markers for all analyzed samples when compared to the cell lysate. With CD81 as an endosomal membrane protein (Escola et al. 1998) and Tsg101 being part of the endosomal sorting complex required for transport (ESCRT) (Katzmann et al. 2001), the results demonstrate that exosomes were supposed to be present in all the samples. However, the exact abundance of both marker proteins varied between the samples with a strong enrichment for lower speed centrifugation, ultracentrifugation and PEG precipitation, while the signal in the NaOAc-precipitated and ExoQuick-TC™ samples was less strong, again for both markers. This matching marker strength suggested the absence of major exosome subpopulations distinguishable by CD81/Tsg101 abundance.

### EV and host cell protein sampling from batch processes

**Fig. 2:**
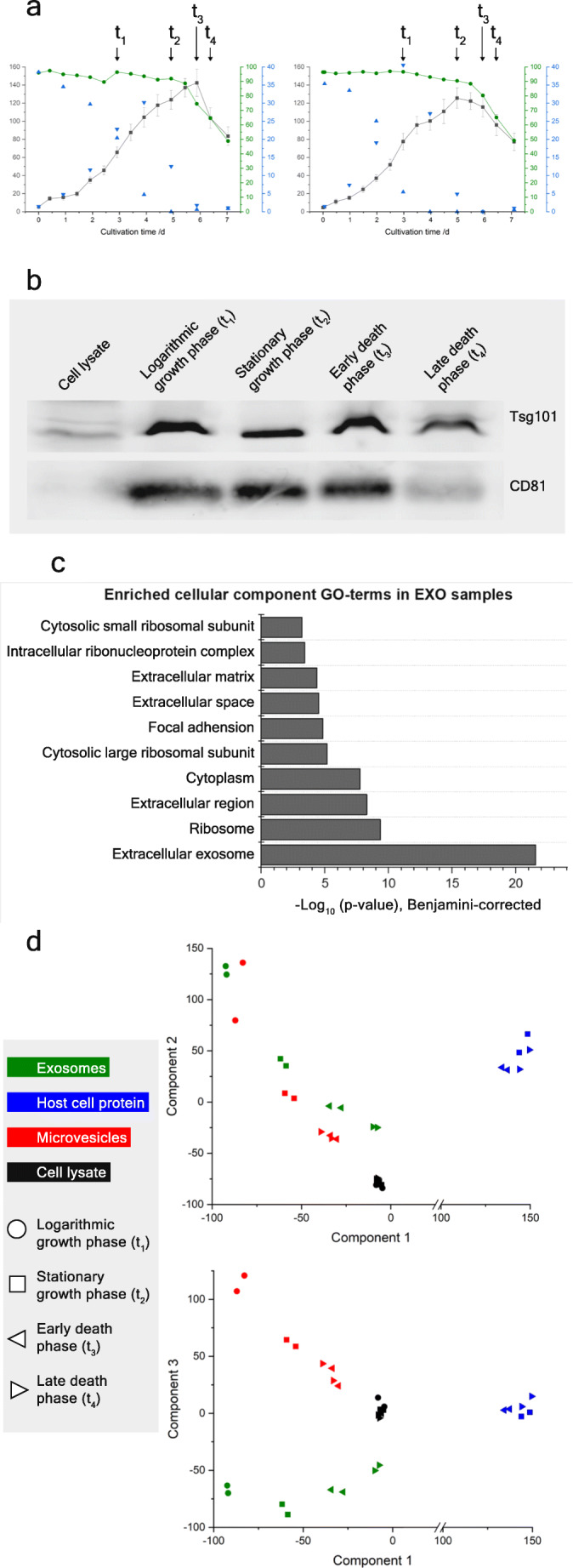
**a** Cultivation data of runned batch processes with viable cell density (black in 1E5 cells per ml), viability (green in %), glucose- and lactate concentrations (blue in mM). Samples were drawn in the logarithmic growth phase (t_1_), stationary phase and twice during the death phase at about 80 (early death phase) and 60% viability (late death phase). **b** Western blot with exosome samples from different growth phases and CHO cell lysate against Tsg101 and CD81. Same protein amounts (20 μg) were loaded for each lane. **c** Bar chart with significantly enriched gene ontology terms of detected proteins in the exosome-derived samples. Calculation was done with DAVID, setting the cell lysate measurements as background and a false discovery rate-threshold of 0.05. **d** Principal component analysis, with clustering of the samples by protein intensity. Components 2 and 3 are plotted against component 1, respectively. Proteins were filtered for > 70% valid values. Host cell protein samples from logarithmic phase were not used for clustering due to very few protein identifications (< 300). Legend: Black = cell lysate; red = microvesicles; green = exosomes; blue = host cell protein; circle = logarithmic growth phase; square = stationary growth phase; triangle, showing left = early death phase; triangle, showing right = late death phase

Because the ultracentrifugation protocol yielded mainly vesicle-like structures with fitting size distribution in transmission electron microscopy and a strong marker enrichment in western blot, we chose this—widely used—method to further characterize the CHO exosome composition over the course of a batch process in a lab-scale bioreactor. Therefore, two parallel bioprocesses in 2 L bioreactors were run. Samples were taken during logarithmic and stationary phase, as well as twice during the death phase at approximately 80 and 60% viability (cf. Fig. 2a), henceforth referred to as early and late death phase. Subsequently, exosome fractions were separated from the culture broth to be analyzed for their proteomic and ncRNA compositions. For comparison, microvesicles, soluble host cell protein, and cell lysate fractions were gathered simultaneously as described in the methods.

### Distinction of the exosome fractions

To validate the origin of exosome samples from the different growth phases, western blots against CD81 and Tsg101 were performed. They showed marker enrichment versus cell lysate at all sampling times (cf. Fig. 2b). While the lower signal for both markers in the later death phase indicates a decreased purity, exosome markers were still present and enriched compared to cell lysate.

For further characterization, mass spectrometry measurements were performed with abovementioned cellular and extracellular fractions from all growth phases, to shed light on the exosomal composition in particular. The samples were measured with the same 1 h liquid chromatography gradient and subsequent data-dependent acquisition mass spectrometry to ensure comparability. A gene ontology enrichment analysis was performed using the proteome data of all exosome samples with cell lysate as background to see which terms are enriched in the exosome samples. Figure 2c plots the significantly enriched gene ontology terms for cellular component according to their −log10 *p*-value (Benjamini-Hochberg corrected). As to be expected, “extracellular exosome” was the most significantly enriched term by far (*p*-value < 10E-20). Supplemental Table S1 lists all proteins with this gene ontology term (558 in total) we detected in our CHO exosome samples. Other prominently enriched terms were “focal adhesion,” as well as “extracellular matrix,” “-space”, and “-region.” Four ribosome-associated gene ontology terms were also among the enriched.

Additionally, overall similarities between all fraction samples from all growth phases were visualized via a principal component analysis in Fig. 2d. It was conducted only with the label-free intensities of all proteins that were detected in more than 70% of the samples. The plotted components C1 to C3 account for a total of 70.1% of explained variance. While the cell lysate samples clustered together across the growth phases on all axes, the host cell protein samples also clustered closely but were distinctly isolated in the most important dimension (component 1), standing for a disparate proteomic composition. Interestingly, exosomes and microvesicles cluster relatively close for the first two components, marking the importance of growth phases for the EV compositions. Moreover, they tend to cluster closer towards the cell lysate as the cultivation proceeds to stationary and death phase, respectively. As for the similarities between exosomes and microvesicles, their effective separation—and therefore composition—is a subject of ongoing debate. Here, we cannot exclude minor cross contaminations, although the vast majority of exosomes should not sediment at a 30 min 20,000*g* centrifugation. In fact, the third component of the principal component analysis in Fig. 2d showed that both fractions indeed formed non-identical populations.

### Proteomic cargo of CHO exosomes

**Fig. 3:**
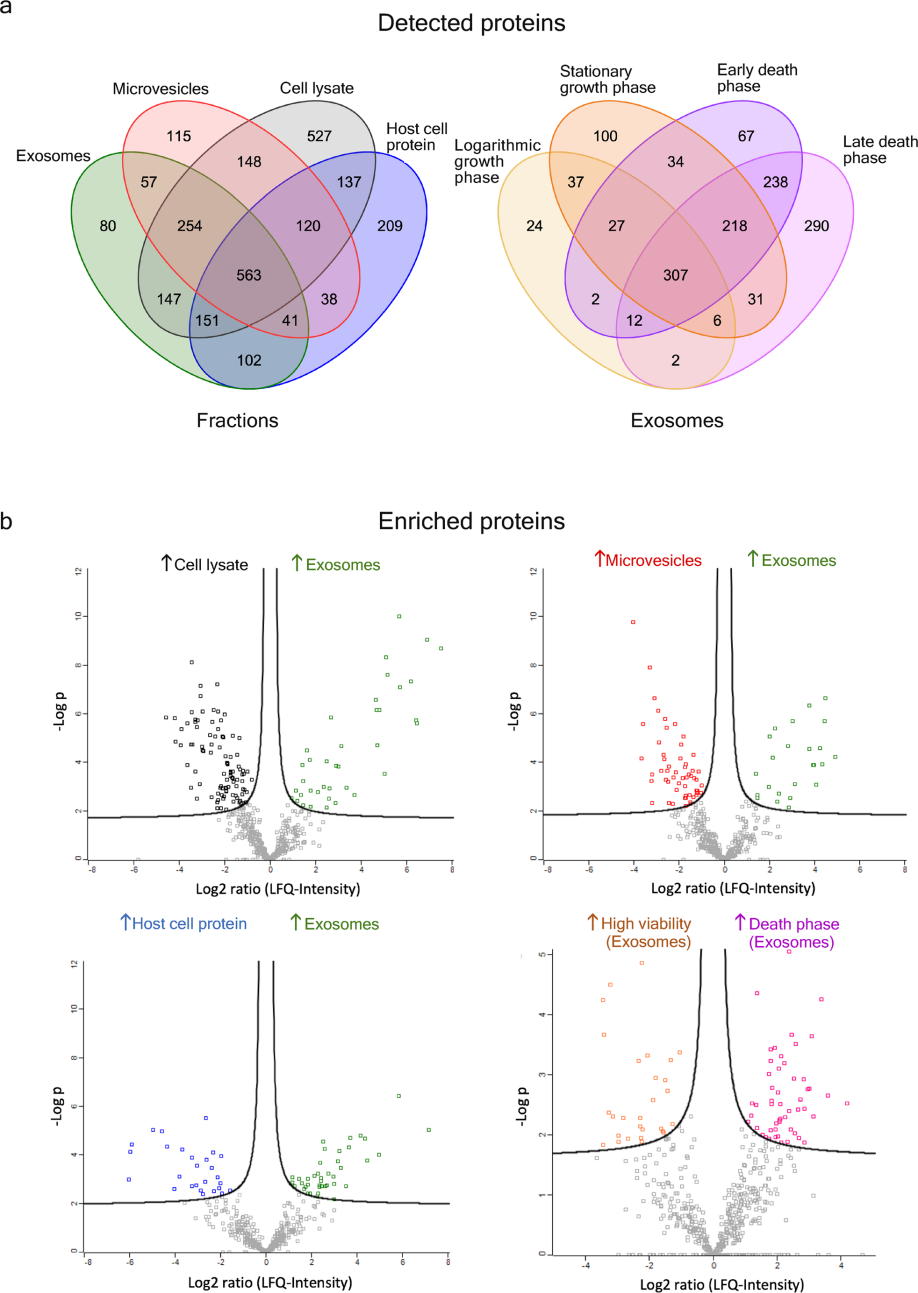
**a** Venn diagramms with detected proteins in different fractions separated from CHO-K1 cells and differentially found species in exosomes from four cultivation phases. Fractions include cell lysate, soluble host cell protein, microvesicles, and exosomes. The compared growth phases are logarithmic phase, stationary phase, as well as early death phase and late death phase. **b** Vulcano plots with significantly enriched proteins per fraction and growth phase. Protein label-free quantification intensity (LFQ) ratios are testes via *t*-test and hyperboles indicate significance thresholds (*p*-value < 0.05, S0 = 0.1, *n* = 8). Exosome fraction was tested against cell lysate, microvesicles and host cell protein. Moreover, exosome compositions from high viability samples (> 95%, logarithmic and stationary phase) vs samples from death phase were compared (*t*-test, *p*-value < 0.05, S0 = 0.1, *n* = 4). The underlying data are listed in Supplemental Tables S2 and S3

Using mass spectrometry-based shotgun proteomics, a total of 1395 proteins were detected in the exosome fractions, 1361 in the host cell protein fractions, 1336 in the microvesicle fractions and 2047 in the cell lysate. Figure 3a depicts the detected proteins in the respective fractions (exosomes, microvesicles, host cell proteins and cell lysate) and the other breaks up the exosomal proteins according to the growth phase they were detected in. In the exosome samples, 80 unique proteins were identified, as well as 115 in the microvesicles and 209 in host cell protein samples. Over the course of the batch cultivation, the number of detected proteins in the exosome samples steadily rose with cultivation time. The late death phase samples displayed the largest share of uniquely detected proteins in the exosome samples (290 proteins) and the two samples from the death phase shared the most similarity in commonly detected proteins, indicating a noticeable influence of apoptosis, be it through changes in exosomal protein composition or apoptosis-derived vesicles.

In Fig. 3b–e, the log2-transformed protein intensities from the exosome fractions were plotted against the respective intensities in the other fractions to check for significantly enriched proteins. Supplemental Table S2 presents a detailed list of all proteins enriched in the exosome fractions compared to the other fractions. Generally, lysosomal proteins such as cathepsin were enriched, indicating its endosomal origin (cf. Fig. 3b, Supplemental Table S2). This was underlined by other proteins that play a role in the multivesicular body biogenesis. Particularly, tetraspanin, vacuolar protein sorting-associated protein 1 (Vta1), and programmed cell death 6-interacting protein (PDCD6IP, also known as Alix) were found to be enriched in the exosome samples. Additionally, different subunits of the T-complex protein 1, which is part of the chaperonin-containing T-complex (TRiC) (Freund et al. 2014) and may be involved in the intracellular vesicle transport (Seo et al. 2010), were enriched. Lamb1 was strongly and consistently enriched in exosomes against all other fractions. Other proteins that seem enriched vs cell lysate, host cell protein and microvesicles were, e.g., peroxidasin and T-complex protein (subunit gamma). Among the proteins enriched in exosomes we also found many that are either part of the extracellular matrix, like collagens or heparan sulfate proteoglycan, and/or interacting with it in some way, like galectin, fibronectin, laminins, or intercellular adhesion molecule 1 (cf. Supplemental Table S2).

Comparing exosomes with microvesicles, proteins associated with multivesicular body formation were enriched in exosomes (cf. Fig. 3c, Supplemental Table S2). Among these are, e.g., Vta1, valosin-containing protein, and clathrin. This underlines the distinct biologic background of microvesicle and exosome fractions, although an utterly complete separation should not be assumed without the application of affinity-based methods. Against host cell protein, mainly extracellular matrix proteins are enriched in exosomes, reflecting the non-membranous nature of the host cell protein samples (cf. Fig. 3d, Supplemental Table S2). However, there are also the histones 1 and 3 that seem to be more abundant in the exosomes.

Figure 3e shows protein enrichments between the exosome samples from different growth phases. While differences between the samples of logarithmic and stationary growth phase and between the death phase time points were rather small (data not shown), differences were large when the high viability samples (logarithmic and stationary phase) were tested against samples from the end of cultivation with viabilities ≤ 80% (cf. Supplemental Table S3). Here, about 80 differentially expressed proteins could be identified. A large proportion of the proteins enriched in the logarithmic and stationary phase were signaling and extracellular matrix proteins, while in the death phase endoplasmic reticulum (ER) and cytoskeleton proteins were more abundant in exosomes. On the other hand, no proteins from the ER or cytoskeleton were enriched in earlier growth phases. Additionally, eleven ribosome-associated proteins as well as 16 chaperones and stress response proteins are enriched in the exosome samples from the death phase compared to high viability earlier growth phases.

### Detected RNA species in the exosome fractions

Next generation sequencing of RNA derived from exosome and cell lysate samples of the same batch process yielded between 31,784 and 126,763 mapped reads for the exosome samples and between 88,322 and 540,453 mapped reads for the cell lysate samples. Although the sequencing was performed only with 15–40 nt long RNAs (137–162 nt with sequencing adapters) which were extracted from a gel to focus on small regulatory RNAs, many of the sequences could be mapped to either mRNA or rRNA. More precisely, between 80% (logarithmic phase) and 40% (late death phase) of the sequences were mapped to mRNAs. For rRNAs, the tendency was less pronounced with 20–30% of the RNA species in the late death phase samples and between 10 and 12% in logarithmic growth phase. However, the data from ribosomal and messenger RNA mappings should be taken with great caution because they are much longer on average—up to several thousand nucleotides—and only fragments of these RNA species were detected in our setup.

### Non-coding regulatory RNA species

**Fig. 4:**
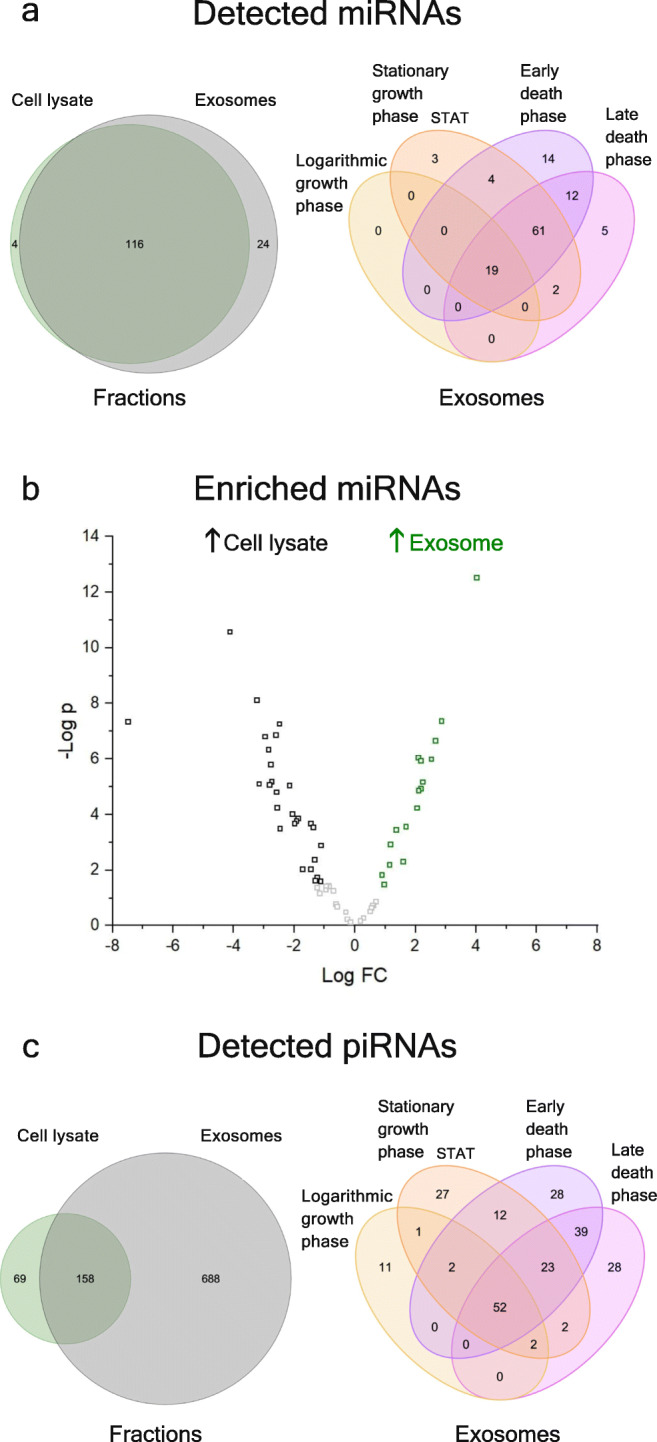
**a** Venn diagramms with detected miRNAs in different fractions separated from CHO-K1 cells and differentially found species in exosomes from four cultivation phases. Fractions include cell lysate, soluble host cell protein, microvesicles, and exosomes. The compared growth phases are logarithmic phase, stationary phase, as well as early death phase and late death phase. **b** Vulcano plots with significantly enriched miRNAs. Normalized miRNA data were tested via *F*-test. Exosome fraction was tested against cell lysate, microvesicles, and host cell protein (*p*-value < 0.05, false discrovery rate < 0.05, *n* = 8). Additionally, exosome compositions from high viability samples (> 95%, logarithmic and stationary phase) vs samples from death phase were compared (*p*-value < 0.05, false discovery rate < 0.05, *n* = 4). **c** Venn diagramms with detected miRNAs in (extra-)cellular fractions (i.e., exosomes, microvesicles, host cell protein, cell lysate) from CHO-K1 cells and differentially found miRNA species in exosomes from different cultivation phases

In all samples, a total of 144 miRNAs were identified. Thereof, 120 were found in exosomes and 140 in cell lysate (cf. Fig. 4a). Of the 120 exosomal miRNAs, 107 (89.2%) had human orthologs listed in the ExoCarta database. Four miRNAs were only present in exosomes, while the vast majority was either detectable in both, cell lysate and exosomes (116 miRNAs) or in cell lysate only (24 miRNAs). The miRNAs only detectable in exosome samples were miR-126a, miR-361, miR-377, and miR-3102. Across the growth phases, 19 miRNAs could be detected in exosomes from the logarithmic phase, 89 in stationary phase, 110 in early, and 99 in late death phase. Three miRNA species were found consistently across all growth phases, miR-29a, miR-378, and miR-92a. miR-24 was detected in all exosome samples from stationary phase onwards. Supplemental Table S4 gives a more detailed overview over the particularly identified exosomal miRNA species, in which growth phase they were found and whether they are significantly enriched or depleted against cell lysate.

When analyzing miRNA abundance, 44 miRNA species were specifically enriched or depleted in exosomes, with 17 significantly enriched in exosomes and 27 in cell lysate (cf. Fig. 4b, Supplemental Table S4). Thereof, members of the let-7 family, a miRNA-family conserved across vertebrates, were exclusively enriched in cell lysate, i.e., significantly depleted in exosomes.

Finally, another non-coding RNA species was detected, namely piRNA. Interestingly, a considerable amount of RNA sequences could be mapped to piRNAs, using the predictions from a previous study (Gerstl et al. 2013). They amounted to up to 25% of mapped RNA sequences, although varying strongly across the samples. From the predicted piRNAs, a total of 227 sequences could be detected in the exosome samples, with 69 piRNAs being exclusively found in this fraction. In contrast, a total of 846 piRNA were detected in cell lysate samples, with 688 of them being specific for cell lysate (cf. Fig. 4c). Compared to cell lysate, six piRNAs (piR-8, piR-23, piR-16474, piR-16475, piR-16480, piR-16483) were significantly enriched in exosomes, while 42 piRNAs were enriched in cell lysate (data not shown). When focusing on bioprocess phases, a subset of 68 piRNAs was identified in exosomes from the logarithmic phase, 121 in the stationary phase, 156 in early, and 146 in late death phase (cf. Fig. 4c).

## Discussion

### Ultracentrifugation yields higher purity than precipitation methods

The current study focused on establishing both suitable exosome separation methods for CHO cells, as well as providing proteome and transcriptome data as initial references to enable further detailed studies on CHO exosomes. Among the separation methods tested, the classic ultracentrifugation protocol showed the best results in transmission electron microscopy, though some vesicles were slightly larger than 150 nm. This indicates that mostly exosomes were present, while some minor contaminations of microvesicles < 220 nm could not be excluded. However, the size determination of the fixed artifacts via electron microscopy may be slightly misleading regarding the native, spherical size of the vesicles (Jung and Mun 2018).

Other protocols resulted in more polydispersity and/or a cloudy smear, which was distinct from crystalline branched stain precipitations and therefore probably derived from supernatant protein. Additionally, in the ExoQuick-TC™ sample small granular aggregate-like looking structures with roughly 30 nm or less in diameter are scattered across the picture. Their shape is distinct from the shrinked cup-shape of vesicles after fixation, so they may lack a lipid bilayer and be protein-derived, non-vesicular contaminants instead. These could be part of a newly described nanoparticle population, also called exomeres (Zhang et al. 2018). Although precipitation-based protocols (ExoQuick-TC™ and NaOAc) yielded the highest amounts of protein, this does not necessarily correspond to higher vesicle recovery, but can as well be a sign of non-EV contaminants. This was supported by appearance of smear and aggregates in the respective electron microscopy pictures. Moreover, the NaOAc precipitation led to the least distinctive enrichment of exosome markers in western blot, endorsing the assumption that its high protein yield is in part due to co-precipitated, non-exosomal contaminants. Since the ultracentrifugation method looks superior in transmission electron microscopy and displayed strong enrichment of marker proteins in western blotting, this method was used for all further experiments.

### Marker proteins in the exosome fractions

In subsequent experiments, comparing exosomes from different growth phases of a bioprocess with other cellular and extracellular fractions, we found endosomal and extracellular matrix-associated proteins like Alix, Vta1, and Lamb1 to be enriched in the exosome fractions. In addition, the gene ontology enrichment analysis rendered “extracellular exosome” and other extracellular terms significantly enriched, underlining these findings and verifying that CHO exosomes were successfully purified. Apart from the endosomal-associated proteins, the enrichment of extracellular matrix proteins could be an effect of the increased surface-to-volume ratio of exosomes compared to cells. We could not find some of the typical exosome markers like CD9 and CD63 in our mass spectrometry database searches, even though we showed, e.g., that CD81 is enriched in our exosome preparations via western blot. We assume that, because of the scarce and mostly unreviewed annotation of *C. griseus* proteins, our proteome data reflect only a part of the presumably present proteins.

### Histones and increasing share of ribosome, ER proteins found in later bioprocess phases

In comparison with extracellular host cell protein samples, we detected histone proteins to be significantly enriched in our exosomes. Although histones as DNA-binding proteins are not to be expected in the supernatant fraction, it is still debated if exosomes actually contain DNA and histones, maybe even use the exosomal secretion for cellular DNA homeostasis (Takahashi et al. 2017), or if the finding of histones is a mere sample impurity derived from dead host cells (Jeppesen et al. 2019).

The longer the cultivation time, i.e., the later the growth phase and lower the viability, the more ER, cytoskeleton, chaperone, and ribosomal proteins we detected in the exosome samples. Exosomes are known to reflect the cellular state (Meldolesi 2018) and therefore, a more complex composition of exosomes may occur over the course of the cellular growth phases. While chaperones apparently represent the cellular reaction to high cell densities, respective nutrient depletion and cellular stress in general (Stacchiotti 2019), the shift in ER and cytoskeleton protein abundance indicates an increasing share of components from other cell organelles in the exosome fractions from the later growth phases. In fact, an increasing similarity of the exosome samples and cell lysate can also be seen in the principal component analysis depicted in Fig. 2d.

Though RNA-binding proteins have repeatedly been reported in exosomes, with ribosomes among them (Keerthikumar et al. 2016), it is interesting to see a difference in their abundance during bioprocessing with increasing shares in later growth phases. Ribosomal RNAs might also be the result of co-purification of 110S ribosomes, since for rat and hamster cells, these ribosome dimers, sizing 50–60 nm in diameter, were reported to form during nutrient starvation (Krokowski et al. 2011). However, due to the overall higher number of detected proteins in the early and late death phase samples, ribosomal enrichment in these samples could also be an artifact.

### Differentially detected miRNA species in exosome samples

The miRNA data indicate that some miRNA species might be preferably loaded or depleted in CHO exosomes, which was described before, e.g., for human embryonic kidney cells (Guduric-Fuchs et al. 2012). Among the miRNA species enriched in exosomes, miR-24 is an interesting finding, because it was previously described as causing growth-inhibition and being overexpressed in stationary phase cells after temperature shift (Gammell et al. 2007). We could detect this miRNA in all exosome samples from stationary phase onwards, which again strengthens the probability for its functional role in CHO exosomes. miR-29a is described in literature as upregulated in CHO cells during exponential growth phase (Klanert et al. 2016), which is what we also observe as an enriched presence in the respective exosome samples. However, others also claim a growth-inhibitory effect of this miRNA for CHO cells (Bort et al. 2012; Muluhngwi et al. 2017). Similar applies to miR-378, which we found to be enriched in CHO exosomes and that is presumably growth-inhibitory species, since its depletion can increase growth and cell density of CHO cells (Coleman et al. 2018; Costello et al. 2018). Therefore, the intracellular depletion via exosomes on the one hand and the intercellular transport and exchange via exosomes of these two growth inhibitory species would make them interesting targets for a rational cell line engineering approach. miR-92a, on the other hand, was also enriched in exosomes and is associated with an increased productivity (Jadhav et al. 2014; Loh et al. 2017). Other potentially interesting miRNAs that were previously studied in CHO cells are miR-744 and miR-3074. miR-744 was reported as a pro-apoptotic miRNA (Kleemann et al. 2018), and indeed we only detected it in our death phase samples. Significantly enriched in exosomes vs cell lysate, miR-3074 was shown to increase recombinant protein production (Weis et al. 2018). On the other hand, let-7 miRNAs, that we found exclusively enriched in cell lysate vs exosomes, are generally regarded as tumor-suppressive and the several subtypes are involved in a plethora of development and differentiation processes.

Although the interest in miRNAs as cell line engineering tools was growing during the past decade (Strotbek et al. 2013; Kelly et al. 2015) and screenings to find potential candidates for improved productivity performed (Jadhav et al. 2012; Fischer et al. 2014), many miRNAs are still not yet characterized in CHO cells and further investigations will be necessary to shed light on the biological relevance of our findings. This applies even more so to piRNAs. Since their function is only poorly researched yet—in fact not at all in CHO—it is difficult to interpret the biological scope of the detected piRNAs. It is interesting, however, that piRNAs are abundant not just in CHO cells but also in secreted exosomes. So far, their existence in exosomes was mainly described in human, diagnostic contexts (Jain et al. 2019; Kolenda et al. 2020). We hope the given data might serve as a reference point for further research in the field of piRNA in CHO cells in general and their exosomes in particular.

### Outlook

Since there is no gold standard for exosome separation, researchers use countless different protocols for exosome purification that fit with their research questions and laboratory equipment. We show here that, while all demonstrated methods yielded exosome-like samples, marker enrichment and EV appearances as well as their size differed clearly, especially between different separation principles. Showing for the first time the differential occurrence of 1395 proteins, 144 miRNAs, and 914 piRNAs across different extracellular fractions and growth phases, the implemented characterization data of the CHO secretome can hopefully prove useful to interested researchers in the field. Overall, we think that the data provide a comprehensive insight into the protein and RNA composition of CHO-derived exosomes, providing many tip-offs for further studies elucidating desired and undesired functions and effects of exosomes in CHO platforms—be it as a growth optimization factor, bioprocess monitoring parameter, or simply as downstream-processing interference.

## Supplementary Information

ESM 1 (PDF 527 kb)

## Data Availability

Mass spectrometry data are available via ProteomeXchange with identifier PXD018446. miRNA and piRNA sequencing data are available at ArrayExpress with accession E-MTAB-9894.
