# Supplementary material for: Exploring the molecular content of CHO exosomes during bioprocessing
Source: Appl Microbiol Biotechnol. 2021 May 3;105(9):3673–89. doi: 10.1007/s00253-021-11309-8 (PMC8102462; doi:10.1007/s00253-021-11309-8)
Supplement: Supplementary file 1 — (PDF 527 kb) [file 253_2021_11309_MOESM1_ESM.pdf]

Applied Microbiology and Biotechnology

# Exploring the molecular content of CHO exosomes during bioprocessing

Christoph Keysberg[1,2], Oliver Hertel[1,3], Louise Schelletter[1,3], Tobias Busche[3], Chiara Sochart[1], Jörn Kalinowski[3], Raimund Hoffrogge[1,3], Kerstin Otte[2], Thomas Noll[1,3].

1) Bielefeld University, Universitätsstraße 25, 33615 Bielefeld, Germany

2) University of Applied Sciences Biberach, Hubertus-Liebrecht-Straße 35, 88400 Biberach, Germany

3) Center for Biotechnology (CeBiTec), Bielefeld University, Universitätsstraße 25, 33615 Bielefeld, Germany

Corresponding author: Christoph Keysberg, tel: +49 151 28888657, fax: +49 7351 582 469, mail: keysberg@hochschule-bc.de,

Table S1: Accession numbers (UniProt ID) and gene names of detected protein groups in CHO-K1 exosome samples that were annotated with the GO term "extracellular exosome".

| **Majority protein IDs** | **Gene names** |
|---|---|
| A0A061HTB4; P14131; A0A061HWF5; G3HJD8; G3I5Y2 | *H671_2g5630; I79_010782; I79_018893; Rps16* |
| A0A061HTC2 | *Actn4* |
| A0A061HTF9; P97461 | *Rps5* |
| A0A061HTH1; Q923D2 | *Blvrb; H671_21760* |
| A0A061HTJ0; P62317; G3HPF1 | *Snrpd2* |
| A0A061HTU1 | *Sbsn* |
| G3HNP4; A0A061HTZ6; P35550 | *Fbl; Fbll1* |
| A0A061HU25; A0A061HUL1 | *Flna* |
| A0A061HU29; A0A061HUL7; G4Y369; G3IBL4; Q00612 | *G6pdx; H671_xg20764; I79_021040* |
| A0A061HU34; P41105; G3HYH7 | *I79_003099; Rpl28* |
| G3H7T9; A0A061HV17; A0A061HUB5; Q7TMY8 | *Huwe1; I79_006430* |
| P0DP28; P0DP27; P0DP26; A0A061HUH1; G3I5T9; G3HBG8; G3IM13; G3I9A7; Q9D6P8 | *Calml3; H671_21721; I79_007796; I79_018847; I79_020143; I79_024941* |
| A0A061HUN3; P62702; G3HPI0; G3HMW2 | *I79_022364; Rps4l; Rps4x* |
| A0A061HUZ2; G3I0M2 | *I79_016910; Pafah1b3* |
| A0A061HVB5; G3H5Y9 | *Tspan6* |
| A0A061HVT8; Q9CZX8 | *I79_010618; Rps19* |
| A0A061HW17; G3H604 | *Gla* |
| A0A061HWC7; A0A061I1J1; G3IBY4; A0A061HVC5; Q99K51 | *H671_xg19868; I79_000762; I79_021170; Pls1; Pls3* |
| A0A061HWR2; Q921Y0; Q8BPB0; G3I4B9; A0A061II12 | *H671_1g3374; Mob1a; Mob1b* |
| A0A061HWY5; G3H9Z3; A0A061ICA1; Q91YP3 | *Dera; H671_4g11415; I79_007226* |
| A0A061HX79; G3GVN0; Q9Z2M7 | *Pmm2* |
| G3HX84; A0A061HZ06; A0A061HXN7; CON__Q3SX14; P13020 | *Gsn* |
| A0A061HXZ8; P06745 | *Gpi* |
| A0A061HYW2 | *Capns1* |
| G3I0R3; A0A061HYX2; A0A061I092; Q99KQ4 | *Nampt* |
| G3H4Z5; A0A061HZ51 | *Mthfd1* |
| A0A061I2E1; A0A061I019; Q9QUM9 | *H671_6g16071; Psma6* |
| G3H5W4; A0A061I021; P62830; G3I5G8 | *H671_7g18546; Ptchd2; Rpl23* |
| G3I5Q9; A0A061I041; P46638; G3I0B0; P62492 | *Rab11ab* |

| | |
|---|---|
| A0A061I094; G3I278; A0A061I6V4; P02469 | *Lamb1* |
| A0A061I0G5; P09103 | *P4hb* |
| G3I1P5; A0A061I0I3; Q9JJU8 | *H671_xg20330; Sh3bgrl* |
| G3I979; A0A061I1P3; P62852; G3I364; G3IFQ9; G3IDG5 | *I79_002588; I79_018003; I79_021743; I79_022582; Rps25* |
| A0A061I1Q2 | *Pros1* |
| G3IDU7; A0A061I2K0; A0A061I3X3 | *Gusb* |
| G3IID2; A0A061I2K9; A0A061I4Z7 | *C1qtnf5; H671_4g13117* |
| G3HFZ7; A0A061I2N8; P49182 | *Serpind1* |
| A0A061I3N6; O70591; G3HU10; A0A061I4J0 | *H671_5g13754; I79_014378; Pfdn2* |
| G3HSH4; A0A061I3X8; Q8BP67 | *I79_021019; Rpl24* |
| A0A061I456; Q9WTM5; G3HZY1 | *I79_005238; I79_016646; Ruvbl2* |
| A0A061I8U4; A0A061I498; A0A061I5J1; A0A061I7X2; Q811D0; G3GSJ8 | *Dlg1; Dlg2; I79_000613* |
| A0A061I4E3; P24547 | *Impdh2* |
| A0A061I4R8; G3HA24 | *Hspb1* |
| A0A061I510; A0A061I766; G3I0Q8 | *Myo6* |
| A0A061I523; Q61398 | *Pcolce* |
| A0A061I5D1 | *H671_1g3310; H671_1g3745; H671_5g13878; Hspa8; I79_022977; I79_024708* |
| G3HFP1; A0A061I5M2; P26043 | *Rdx* |
| A0A061I677; G3HKQ7 | *I79_011286; Map4* |
| G3I5H1; A0A061I6A0; P24472 | *Gsta1; Gsta3; Gsta4; I79_018720* |
| A0A061I6G9; O55131; G3HTJ2; A0A061I443 | *H671_4g12516; H671_4g12857; I79_014218; Sept7* |
| A0A061I7K3; A0A061I6R1; G3H6B2; P39054 | *Dnm2; H671_4g12320; I79_003271; I79_005867; I79_021012* |
| G3HVR6; A0A061I7S0; A0A061I6X6; Q9R0B9 | *H671_4g12243; I79_015057; Plod2* |
| G3GU73; A0A061I725; Q6PHN9 | *Rab35* |
| G3H8F9; A0A061I739; Q9QUI0 | *4930544G11Rik; H671_4g12612; Rhoa* |
| A0A061I743 | *Ahnak* |
| A0A061I7K0; G3H6B7 | *Slc44a2* |
| A0A061I835; G3GWF4; Q61699 | *Hsph1* |
| G3H453; G3I1I8; A0A061I856; P27048; P63163 | *Snrpb; Snrpn* |
| G3IHN6; O55221; A0A061I8B2 | *Slc3a2* |
| A0A061I8C8; G3I7M2; A0A061IAR7; Q8BFR5 | *I79_002296; Tufm* |
| A0A061I8K5; G3I5F0; O88545 | *Cops6* |
| G3H3P8; A0A061I8U9 | *Hexa* |
| A0A061I8W9; G3HCX3 | *Dnase2* |
| G3GV75; A0A061I9V4; P63321 | *H671_3g11115; Rala* |
| A0A061IA65; G3H289; P47738 | *Aldh2; H671_4g11405* |

| | |
|---|---|
| A0A061IAR2; A0A061I412; A0A061I8K0; A0A061I7N8; A0A061I5B5; G3HH34 | *Col12a1; H671_4g11986; I79_009930* |
| A0A061IAT0; Q9R0E1; G3HA51 | *H671_4g11939; Plod3* |
| G3I4I9; A0A061IB55; Q9EQK5 | *Mvp* |
| G3HT77; A0A061IBA1; Q7TMB8; G3I4G5; Q5SQX6 | *Cyfip1; H671_3g9001; I79_018344* |
| A0A061IBI6; Q8VDM4; G3I310 | *I79_017808; Psmd2* |
| A0A061IBK9; G3GT06 | *Cct8* |
| A0A061IBY9; Q9CYL5 | *Glipr2; H671_2g7362* |
| A0A061ICB3; G3HCV4; A0A061IEI3 | *Man2b1* |
| A0A061ICE4; Q03265 | *Atp5a1* |
| A0A061ICG7; G3GX17; Q6PB93 | *Galnt2* |
| A0A061ICH9; G3I3H9; A0A061IEN5; Q922Q9 | *Chid1; H671_3g9873* |
| G3I740; A0A061ICY3 | *Vnn1* |
| A0A061ID29; Q64433 | *H671_2g6480; Hspe1; I79_011397* |
| G3I064; A0A061IF92; A0A061ID46 | *Ganab* |
| A0A061ID55 | *Ahnak* |
| A0A061ID82; Q8CDN6; G3H5G6 | *Txnl1* |
| A0A061IE32; G3HL13; P63038 | *H671_6g16037; Hspd1* |
| G3I412; A0A061IE40; A0A061IKQ5; A0A061IF41; Q61112 | *Sdf4* |
| G3HCW3; A0A061IEH0; O54984 | *Asna1* |
| A0A061IL44; A0A061IJ24; A0A061IEH1; A0A061IFH1; G3IH63 | *H671_2g5970; Myh9* |
| G3IMH4; A0A061IEQ5 | *Smpd1* |
| G3I3B5; A0A061IFC2; P84096 | *H671_3g8575; Rhog* |
| A0A061IFD0; G3I3B7; P07742 | *Rrm1* |
| G3IJF3; A0A061IGK5; A0A061IM73; A0A061IFK2; Q6P9J9 | *Ano6; I79_023987* |
| A0A061ILI4; A0A061IFX0; Q8BFY9; A0A061IEV8 | *Tnpo1; Tnpo2* |
| G3I0D8; A0A061IGK9 | *Cyb5r3; H671_2g7251* |
| A0A061IGY1; G3H4E0 | *Bst2; I79_005141* |
| G3IF52; A0A061IH04 | *Nucb2* |
| G3HU28; A0A061IH36; A0A061II46 | *Capzb; H671_2g6288* |
| G3I437; A0A061IHM8; A0A061IF33 | *Agrn; H671_2g6103* |
| A0A061IHT5; A0A061IKX4; G3I2D8; A0A061IIW3; A0A061IFA3 | *Epb4.1l2; Epb41l2* |
| A0A061IIB1; G3I3A8 | *I79_017912; Numa1* |
| A0A061IIE7; Q9CXP8 | *Gng10* |
| G3HMB9; A0A061IIJ6; Q3THW5; P0C0S6; G3ILX7 | *H2afv; H2afz; H671_xg19886* |
| A0A061IQ19; A0A061IJ77; G3HRX0; Q6P069 | *Sri* |

| | |
|---|---|
| A0A061IKW7; A0A061IQH5; A0A061INC7; A0A061IP91; G3HWP9; A0A061IJX8; Q9Z204 | *H671_2g6160; H671_2g6266; Hnrnpc* |
| G3H6V7; A0A061IKA1; P11152; Q8R4V8 | *Lpl* |
| G3HUU7; A0A061ILN9; A0A061IKI0 | *H671_1g3382; S100a10* |
| A0A061ILJ8; A0A061IKK9; Q8VDN2; G3H8Z9 | *Atp12a; Atp1a1; Atp1a3; Atp4a; H671_21334; I79_006481; I79_016917* |
| A0A061IKY1 | *Bgn; H671_1g2241; I79_010810* |
| A0A061IL36; A0A061IIK0; G3HNA5; Q9JKB1 | *H671_1g3085; Uchl3* |
| A0A061ILB4; G3IMD1 | *Adh5; Adh7* |
| G3HW44; A0A061ILQ3; Q68FL6 | *Mars* |
| A0A061IM94; G3HWC3; P26040 | *Ezr* |
| G3I5A4; A0A061IML2 | *Anxa5; H671_1g1311* |
| A0A061IN67 | *H671_1g1045; Sema3c* |
| A0A061INU9; P26638 | *Sars* |
| G3HMI3; A0A061IP17 | *H671_1g1046; Sema3c* |
| A0A061IPM3; Q9Z2U1; G3I7A8 | *I79_019395; Psma5* |
| A0A061IQ35; P62814; G3H6W0 | *Atp6v1b1; Atp6v1b2* |
| A0A061IQ65; A0A061IQU0; G3HJS0 | *Sptb; Sptbn1; Sptbn4* |
| A0A061IQB8; P62984; G3GVL0; G3I129; O35079; O35080; P0CG49; P0CG50 | *CHUB2; I79_005093; Kxd1; Ubb; Ubc* |
| G3HC31; A0A061IQJ9; P14069 | *H671_1g1493; S100a6* |
| G3HM98; A0A061IR35 | *Dbnl* |
| A0A061IR66; G3HW36; Q9CZN7 | *Shmt2* |
| G3HC25; A0A061IR84 | *H671_1g1490; S100a13* |
| A0A098KXB1; A0A098KXE1; G3HR08 | *Lap3* |
| A0A098KXC9; Q9WVJ2 | *Psmd13* |
| A0A2Z6LPP5; G3H303; P99026 | *Psmb4* |
| B0V2N1; A0A061I2U3; G3HCI6 | *Ptprd; Ptprs* |
| G3GT56; B2ZA78; A0A061I985; A0A061IBE4 | *Gart; H671_4g11686* |
| G3HZE3; B8Y440; O35657 | *Neu1* |
| CON__P05787; P11679; G3HES2; A0A061I7Q7; CON__Q9DCV7; G3IH98; Q9DCV7 | *Krt7; Krt8* |
| F7J0L2; Q6PDM2; F7J0L3 | *Srsf1* |
| G3GR73; Q61598 | *Gdi2* |
| G3GRM2; O35598; A0A061I1D6 | *Adam10; H671_4g12147* |
| G3GRT0 | *Aprt* |
| G3GRU9 | *Slc7a5* |
| G3GSG4 | *Atp6ap2* |
| G3GSH5; A0A061HYF2; A0A061HXV1; Q62167; P16381; Q62095 | *Ddx3x; H671_5g13982* |

| | |
|---|---|
| G3GSZ1; Q8R4W6 | *Pcolce2* |
| G3GTH2; P70441 | *Slc9a3r1* |
| G3GTS6 | *Psmd11* |
| G3GTX5; Q06335 | *Aplp2* |
| G3GU60 | *Pebp1* |
| G3GU76; P14869; G3HKG9 | *H671_xg20133; I79_008756; I79_013964; Rplp0* |
| G3GUD8 | *Anp32b* |
| G3GUI3 | *Fkbp4* |
| G3GUR1 | *C1ra* |
| G3GUU5; P40142 | *Tkt* |
| G3GVM9; A0A061HW36; Q9CR86 | *Carhsp1; H671_7g18768* |
| G3GVX2; Q9R0P5 | *Dstn* |
| G3GW11; P32020 | *Scp2* |
| G3GW47 | *Rars* |
| G3GWX6 | *Serpinb1a* |
| G3GX44; Q9D1M0 | *Sec13* |
| G3GX55; Q8BKX1 | *Baiap2* |
| G3GX75; A0A061I138; A0A061I3A9; Q99LI8 | *H671_7g18346; Hgs* |
| G3GX96; P59999 | *Arpc4* |
| G3GXB0; Q99PT1 | *Arhgdia* |
| G3GXD7; A0A061I1B1; A0A061I3F6; A0A061HWV7 | *Fasn; H671_7g18362* |
| G3GXS2 | *Emilin1* |
| G3GXT2; B2RQC6 | *Cad; CAD* |
| G3GXZ0 | *Tgm2* |
| G3GY17 | *Cand1* |
| G3GY22; Q99JI6 | *Rap1b* |
| G3GY44; Q61879 | *Myh10* |
| G3GY47; P61255 | *Aox1; H671_3g9631; H671_4g12607; I79_010111; Rpl26* |
| G3GY52 | *Pfas* |
| G3GYJ1; Q5FWK3 | *Arhgap1* |
| G3GYP7; Q9JII6 | *Akr1a1* |
| G3GYP9 | *Prdx1* |
| G3GYR9; P62242; A0A061IP26 | *H671_1g1040; I79_024996; Rps8* |
| G3GYY6; P24270 | *Cat; H671_5g13851; Sarg* |
| G3GYZ1; P15379 | *Cd44* |
| G3GZ56; G3IHR8; Q80SW1; Q68FL4; A0A061IMQ1; A0A061IP70 | *Ahcyl1; Ahcyl2; H671_1g2294; I79_023374* |
| G3GZ85; Q78PY7 | *Snd1* |
| G3GZ95; Q9D1K2 | *Atp6v1f* |
| G3GZB2 | *Asah1* |
| G3GZE6; Q99JY9 | *Actr3; Actr3b* |
| G3GZL7 | *Cacybp* |
| G3GZY4; Q924C6 | *Loxl4* |
| G3GZZ0 | *Got1* |

| | |
|---|---|
| G3H066; P50516 | *Atp6v1a* |
| G3H0C2; Q9R1P0 | *Psma4* |
| G3H0E4 | *Cspg4* |
| G3H0L9 | *Ctsb* |
| G3H0S4 | *Sord* |
| G3H0S7 | *B2m* |
| G3H0U6 | *Pdia3* |
| G3H0X2; Q9D1G5 | *Lrrc57* |
| G3H125; Q80ZC6 | *Slc1a4* |
| G3H154 | *H2afy* |
| G3H1D5 | *Scpep1* |
| G3H1F4; Q9CZM2 | *Rpl15* |
| G3H1K9 | *Actn1; Actn3* |
| G3H1M4; Q6ZWX6 | *Eif2s1* |
| G3H1S2; Q6P5E4 | *Uggt1* |
| G3H1W4 | *Tinagl1* |
| G3H204; Q9ER00 | *Stx12* |
| G3H284 | *Erp29* |
| G3H2C9; Q9WTX5 | *Skp1* |
| G3H2I6 | *Ncam1* |
| G3H2N6; Q9WU78 | *Pdcd6ip* |
| G3H2P3; A0A061I7D9 | *Glb1; H671_4g12443* |
| G3H2T7; G3HPV9; G3HDU8; G3HDS5; G3HDT2; P84228; P68433 | *H2afz; H3f3a; H3f3c; Hist1h3a; Hist1h3b; Hist2h2aa1; I79_004539; I79_006416; I79_010016; I79_013751; I79_014844* |
| G3H2W6 | *Ecm1* |
| G3H352; A0A061HZQ1 | *Dync1h1; H671_5g13885* |
| G3H3E3 | *Cant1* |
| G3H3E4 | *Lgals3bp* |
| G3H3E6; P25785 | *Timp2* |
| G3H3H9; Q8VCT3 | *Rnpep* |
| G3H3I9; P97315 | *Csrp1* |
| G3H3Z5; P61514; A0A061I7S2; G3HWS6; G3H2W7; G3HNA9; A0A061IQN3; A0A061HUX0 | *H671_1g2050; H671_xg20578; Rpl37a* |
| G3H412; P17918 | *Pcna* |
| G3H4D3 | *Pgls* |
| G3H4V1; P42208 | *Se2* |
| G3H515; P61028 | *I79_013748; Rab1A; Rab8b* |
| G3H533; P24369 | *Ppib* |
| G3H559; P27046 | *Man2a1* |
| G3H576; P14824 | *Anxa6* |
| G3H577 | *Gm2a* |
| G3H5A6; G3HPQ9; A0A061HYH8 | *Fth1; H671_2g7222; H671_6g17076; I79_005484* |
| G3H5E5; Q9JIW9 | *Ralb* |
| G3H5W0; Q9R1P1; A0A061I0Q5 | *Psmb3* |

| | |
|---|---|
| G3H638; Q8BK64 | *Ahsa1* |
| G3H6C5; Q61081; A0A061I5Y9 | *Cdc37; H671_4g13050* |
| Q4PNY4; G3H6M5; P97807 | *Fh* |
| G3H6X6 | *Utrn* |
| G3H6Z4; Q8CI94 | *Pygb* |
| G3H7I6 | *Qsox1* |
| G3H7K5; P80316 | *Cct5* |
| G3H7Y0; Q8CIE6 | *Copa* |
| G3H7Z2 | *H671_8g19398; I79_021182; Tagln2* |
| G3H893 | *Abhd14a* |
| G3H894; Q8VCR7 | *Abhd14b* |
| G3H8C9; Q62177 | *Sema3b* |
| G3H8D0; Q3HR13; P08752 | *Gnai2* |
| G3H8F4; Q62165 | *Dag1* |
| G3H8K2 | *Man1a1* |
| G3H8N3; A0A061IIV5; G3HTP4 | *H671_2g6048; Rpl4* |
| G3H8V1 | *Mmp9* |
| G3H8V5 | *Ctsa* |
| G3H8Z6; Q8R143 | *Pttg1ip* |
| G3H945; Q9QZD9 | *Eif3i* |
| G3H9S0 | *Fam129a* |
| G3H9V0 | *Psmb2* |
| G3H9W7 | *Bclaf1; Thrap3* |
| G3HA23; P08249; A0A061I397 | *H671_4g12240; Mdh2* |
| G3HA54; A0A061I8M5 | *H671_4g11943; Serpine1* |
| G3HAN1; Q9D8B3 | *Chmp4b; Chmp4c; H671_6g15512* |
| G3HAP7 | *Gss* |
| G3HAQ2; O55135 | *Eif6* |
| G3HAR2; Q8C166 | *Cpne1* |
| G3HAV4; Q9CR26 | *Vta1* |
| G3HB04 | *Pdia6* |
| G3HB86 | *Rarres2* |
| G3HBI1; Q3UQ28 | *Pxdn* |
| G3HBI9; P24527 | *Lta4h* |
| G3HBP8; P25444; Q6TMK7; G3I7G4; G3HYV9; G3HIC0 | *Gm8225; I79_000574; I79_007512; I79_012597; I79_017124; I79_018431; I79_018578; Rps2* |
| G3HBU2; Q9WUD1 | *Stub1* |
| G3HC01; P21107 | *Tpm3* |
| G3HC47 | *Gba* |
| G3HC49; Q05895 | *Thbs3; Thbs4* |
| G3HCH0; Q9CPT4; A0A061I2T5 | *H671_5g15168; Mydgf* |
| G3HCL6 | *C3* |
| G3HCS4; Q9D8W5 | *Psmd12* |
| G3HCT4; Q61656 | *Ddx5* |
| G3HCW9; A0A061I7R6 | *H671_3g9966; Prdx2* |
| G3HCX8 | *Calr* |

| | |
|---|---|
| G3HD57; O08992 | *Sdcbp* |
| G3HD85 | *Tax1bp1* |
| G3HD97; Q91W90 | *Txndc5* |
| G3HDD4; P29595 | *Nedd8* |
| G3HDE5; G3HUD3 | *Psme1* |
| G3HDQ1; Q91ZJ5 | *Ugp2* |
| G3HDQ2; P14152 | *Mdh1* |
| G3HDT7; Q6ZWY9; Q64478; P10853; G3IB92; G3HDT0; Q8CGP1 | *Hist1h2bc; I79_008696* |
| G3HEE8; P48441 | *Idua* |
| G3HES3; Q9ERK4 | *Cse1l* |
| G3HFF0; G4Y366; P17809 | *Slc2a1* |
| G3HFM4; A0A061IP39; Q80X90 | *Flnb; H671_1g3080; H671_1g3441* |
| G3HG25 | *Lamc1; Lamc3* |
| G3HG36 | *Glul* |
| G3HG83; P80318 | *Cct3* |
| G3HGM6 | *Aga* |
| G3HGP4 | *Acat3* |
| G3HGP6; P11983 | *Tcp1* |
| G3HGW6 | *I79_014076; Lama5* |
| G3HH30 | *Akr1b1; Akr1b3* |
| G3HH63 | *Plaa* |
| G3HHA9; P97384 | *Anxa11* |
| G3HHK3; P62900; G3H7J9 | *I79_003816; I79_006494; I79_023438; Rpl31* |
| G3HHR3; P20152 | *4732456N10Rik; Des; Gfap; I79_023619; Ina; Krt75; Krt83; Krt84; Krt85; Nefh; Nefl; Nefm; Prph; Vim* |
| G3HHR9; A0A061I2I0; P43406 | *Itgav* |
| G3HHY9; Q9CR51 | *Atp6v1g1; Atp6v1g2* |
| G3HIC5; P60122 | *Ruvbl1* |
| G3HID1; P51150 | *Rab7a* |
| G3HIM1 | *Hspg2* |
| G3HIM4; P60766 | *Cdc42* |
| G3HIQ0; Q9D0R2 | *Tars* |
| G3HIQ1 | *I79_001870; I79_022247; Ppia* |
| G3HIS0; Q99JI4 | *Psmd6* |
| G3HIX6; P32921 | *Wars* |
| G3HJM2 | *Gars* |
| G3HJQ8 | *Itga3* |
| G3HK00; P68040 | *Gnb2l1* |
| G3HKG8; P97351 | *H671_1g2169; I79_025236; Rps3a; Rps3a1* |
| G3HKX9; Q80VA0 | *Galnt7* |
| G3HKY3 | *Atp2b1* |
| G3HKZ1; P63101 | *Ywhaz* |
| G3HLK3 | *Grn* |
| G3HLS2; Q9CQV8 | *Ywhab* |
| G3HLT3 | *Sdc4* |

| | |
|---|---|
| G3HMN4; A0A061I7G3 | *Nit2* |
| G3HMV2; Q9CXW4 | *H671_2g6755; I79_015679; Rpl11* |
| G3HMV7 | *Fuca1* |
| G3HN65; Q01730 | *Rsu1* |
| G3HN88; P40124 | *Cap1* |
| G3HN89 | *Ppt1* |
| G3HNG2 | *Acot1; Acot2; Acot6; I79_012304; I79_012305* |
| G3HNJ3 | *Clu* |
| G3HNK4; O08553 | *Dpysl2* |
| G3HNV4; A0A061IJP1 | *H671_2g5691; Tpi1* |
| G3HP24; Q8BU30 | *Iars* |
| G3HP75; Q61753 | *H671_3g10126; Phgdh* |
| G3HPH9; G3INL9; P54751 | *St3gal1; St3gal-1* |
| G3HPZ5 | *Capg* |
| G3HQ45; Q9Z0N1; A0A061HV36; Q9Z0N2 | *Eif2s3x; H671_xg20498* |
| G3HQ69; Q60598 | *Cttn* |
| G3HQD5; P97467 | *Pam* |
| G3HQM6; P08113 | *Hsp90b1* |
| G3HQP8; P99024; Q60454 | *H671_3g8728; Tubb5* |
| G3HQY6 | *Lipa* |
| G3HRD9; O55234 | *Psmb5* |
| G3HRF8; A0A061I987; Q61490 | *Alcam; H671_4g11392* |
| G3HRG8 | *Erp44* |
| G3HRK0; P50580 | *Pa2g4* |
| G3HRN0; P56480 | *Atp5b* |
| G3HRQ4; Q60605; G3HJV4 | *Myl6; Myl6b* |
| G3HSE4; Q9R1P4 | *Psma1* |
| G3HSF3; P49722 | *Psma2* |
| G3HSL4 | *Eef2* |
| G3HSZ4; Q8BL97 | *Srsf7* |
| G3HTE5 | *Gaa* |
| G3HTF8; Q64010 | *Crk* |
| G3HTG9 | *Glod4* |
| G3HTU8 | *Galk1* |
| G3HU13; Q9CR09 | *Ufc1* |
| G3HU24; Q8CG76 | *Akr7a2* |
| G3HU51; O88844 | *H671_1g3584; Idh1* |
| G3HUI4; A0A061ID77 | *Prcp* |
| G3HUM5; A0A061HTI7 | *Gpi; I79_014638* |
| G3HUU6; A0A061II04 | *S100a11* |
| G3HVB8; P63005; A0A061I0R0 | *H671_7g18533; Pafah1b1* |
| G3HVZ2; A0A061IMJ2; A0A061INM1 | *Fat1* |
| G3HW48; A0A061ILP9 | *Dctn2* |
| G3HWB8; P09671 | *Sod2* |

| | |
|---|---|
| G3HWE4 | *Nid1* |
| G3HWJ3 | *Plec* |
| G3HWJ7 | *Scrib* |
| G3HX33 | *Rpl7* |
| G3HX53; Q61009 | *Scarb1* |
| G3HX62; Q9WVM1 | *Racgap1* |
| G3HX83; Q91V41 | *Rab14* |
| G3HXL1; P60335 | *Pcbp1; Pcbp2* |
| G3HXN7 | *Hexb* |
| G3HXW9; P61164 | *Actr1a* |
| G3HXZ3 | *Cd81* |
| G3HY03 | *Ddt* |
| G3HY08 | *Mif* |
| G3HYB7 | *Cct7; H671_2g6488; I79_022232* |
| G3HYT5 | *Dnph1* |
| G3HYW3; A2A432 | *Cul4b* |
| G3HZ28; Q9EP52 | *Twsg1* |
| G3HZ42; P80314 | *Cct2* |
| G3HZ55; P09055 | *Itgb1* |
| G3HZF4; Q9Z1Q5; A0A061IIJ8; A0A061IPN4 | *Clic1* |
| G3HZG2; P67871 | *Csnk2b* |
| G3I000 | *Aldh16a1* |
| G3I004; P62281 | *Rps11* |
| G3I008; Q8VEJ9 | *Vps4a* |
| G3I018; P26516; A0A061IC12 | *H671_3g9708; Psmd7* |
| G3I0C8 | *Acat1; H671_4g12081* |
| G3I0F7 | *Naga* |
| G3I0Q1; P62855; G3H6W7; G3I583; G3INF1; G3HRK1 | *I79_013507; I79_023563; Rps26* |
| G3I1H5 | *Lgmn* |
| G3I1P0; Q91V64 | *Isoc1* |
| G3I1V3 | *Fn1* |
| G3I1V4 | *Atic* |
| G3I1X8; P62874 | *Gnb1* |
| G3I1Y9 | *Psap* |
| G3I216 | *Tpi1* |
| G3I2E9; Q9D1G1 | *Rab1b* |
| G3I2F5; O54962 | *Banf1* |
| G3I2G7; Q9WVJ9 | *Efemp2* |
| G3I2H6; Q8BWP8 | *B4gat1* |
| G3I2I9; P27659 | *H671_1g1861; Rpl3; Rpl3l* |
| G3I2M1; P80317 | *Cct6a; H671_8g19698* |
| G3I2V6 | *Plxnb2* |
| G3I2Y1; A0A061IAK8; P05202 | *Got2; H671_3g9301* |
| G3I390; A0A061I6N9; Q9WV32 | *Arpc1b; H671_4g11473* |

| | |
|---|---|
| G3I391; Q9R0Q6 | *Arpc1a* |
| G3I3F3; A0A061IDQ0 | *H671_3g9396; Rnh1* |
| G3I3G8; Q93092 | *Taldo1* |
| G3I3H2; P99027 | *I79_003400; Rplp2* |
| G3I3K5 | *Gpr56* |
| G3I3U5 | *Nid1* |
| G3I3W4 | *Esd* |
| G3I3Y6 | *Gstp1* |
| G3I3Z5; Q9WUM3 | *Coro1b; Coro6* |
| G3I4H6; A0A061IB69; P05064 | *Aldoa; Aldoc* |
| G3I4W7 | *Ctsd* |
| G3I4Z7; P16045 | *Lgals1* |
| G3I5L3; A0A061I6Z5 | *Anxa1* |
| Q99PF7; G3I5R2; P62858 | *Rps28* |
| G3I692; Q9D1A2 | *Cndp2* |
| G3I6E3; O88952 | *Lin7a; Lin7c* |
| G3I6T1 | *H671_4g11723; Plbd2* |
| G3I7C7; A0A061I910; P70452 | *Stx4* |
| G3I7T9; Q9DAS9 | *Gng12* |
| G3I881; P45878 | *Fkbp2* |
| G3I887 | *Prdx5* |
| G3I936; J9T0K1 | *Npc2* |
| G3I968; P27661 | *H2afx* |
| G3I973; Q9JKR6; A0A061I5C0; A0A061I657 | *H671_4g12994; Hyou1* |
| G3I9G3 | *Cpne2; Cpne3; Cpne5; Cpne7; Cpne8; I79_002323; I79_003239; I79_008556* |
| G3I9L7 | *Efemp1* |
| G3I9X6 | *Sptan1* |
| G3IA32; O88544 | *Cops4* |
| G3IA94; Q3THE2; G3GXV8; Q9CQ19 | *Myl12b; Myl9* |
| G3IAG0; Q6P1B1 | *Xpnpep1* |
| G3IAQ0 | *Eno1; Eno3; H671_1g3671; H671_2g6627; I79_001117; I79_019072* |
| G3IBF7; P61087 | *Ube2k* |
| G3IBG3; Q02053 | *Uba1* |
| G3IBH0 | *Timp1* |
| G3IBI6; P62274 | *Rps29* |
| G3IBK8; Q9R1Q9; A0A061HZ53 | *Atp6ap1* |
| G3IBV7; P56395 | *Cyb5a* |
| G3IC08; Q8R0J7 | *Vps37b* |
| G3ICD3 | *Mfge8* |
| G3ICW1; Q62422 | *Ostf1* |
| G3ICY6; P63276 | *Rps17* |
| G3IDD4; P19324; Q9Z1W7 | *Serpinh1* |
| G3IDE4 | *Tpp1* |

| | |
|---|---|
| G3IDL3; A0A061IAK0; Q9CQW9; Q99J93 | *H671_3g10396; Ifitm3* |
| G3IDM2; P18760 | *Cfl1* |
| G3IDN7; Q91VU0 | *Fam3c* |
| G3IDS2 | *Capza1; I79_007064* |
| G3IDS7 | *Slc16a1* |
| G3IE06; Q922P8 | *Tmem132a* |
| G3IE21 | *Akr1b8* |
| G3IEB7 | *Oaf* |
| G3IEF1 | *Fth1* |
| G3IEG2 | *Serpinb6* |
| G3IEK1; Q9CVB6 | *Arpc2* |
| G3IEU2 | *Park7* |
| G3IEX0; Q3V1T4 | *Lepre1* |
| G3IF62 | *Iqgap1* |
| G3IF80 | *Ncl* |
| G3IFX0; P12815; G3IPL7 | *I79_022645; Pdcd6* |
| G3IG05 | *Anxa2* |
| G3IG23; Q8BGQ7 | *Aars* |
| G3IGZ8; Q9ER41 | *Tor1b* |
| G3IHH6 | *Fah* |
| G3IHQ6 | *Mpst* |
| G3IHY5 | *Pgd* |
| G3IHZ2; Q8VBV7 | *Cops8* |
| G3IIB1 | *Siae* |
| G3IIE7 | *H671_2g6096; Plod1* |
| G3IJV7 | *Acot7* |
| G3IK05 | *Mfge8* |
| G3IKE2; P62245; A0A061IEG2; A0A061IDH1; G3H011 | *H671_21626; H671_3g9123; I79_002179; I79_003459; I79_004126; I79_019722; Rps15a* |
| G3IKQ5; G3I9E7; Q8K183 | *I79_024457; Pdxk* |
| G3IKQ6 | *Cstb* |
| G3IKX2 | *Serpinf1* |
| G3ILF1; A0A061IJD0 | *Gstm5; H671_1g2859* |
| G3IM56; Q8R105 | *Vps37c* |
| G3IN86 | *Dpp7* |
| G3INC5 | *Ctsl* |
| G3IQ06 | *Serpinf1* |
| M1RNR0; M1R995; M1R372; A0A061IRG6; Q9DCL9 | *Paics* |
| M5AJ86; Q5SWU9; G3H9D1 | *Acaca; Acacb; H671_4g13271* |
| O08529; G3GRB1; A0A061I368 | *Capn2; H671_5g15004* |
| O70475; G3INU6 | *Ugdh* |
| O88207 | *Col11a1; Col5a1* |
| Q5VLK2; P05213; G3I6I6; G3HWE9; P68368; G3INA2 | *H671_4g12032; I79_005464; I79_021381; I79_025414; Tuba1a; Tuba1b; Tuba1c; Tuba3a; Tuba4a; Tubal3* |

| | |
|---|---|
| Q540F6; P10126; G3HH39 | *Eef1a1; Eef1a2; I79_009935* |
| P11031 | *Sub1* |
| P11499; G3HC84 | *H671_2g6722; Hsp90aa1; Hsp90ab1; I79_021139; Lad1* |
| P12023; G3HMG4 | *App* |
| P12970; G3I9S3; G3HMP4; G3GSB7 | *I79_000336; I79_006267; I79_011461; I79_013061; I79_013895; I79_014211; I79_014285; I79_015111; I79_018340; I79_019326; I79_020677; Rpl7a* |
| P14148; G3GZ17; A0A061IJF5; G3HX34 | *H671_3g11247; H671_7g18661; Rpl7* |
| P14206; G3HQX0 | *H671_4g12653; I79_013226; Rpsa* |
| P17751 | *Tpi1* |
| Q3HR09; P21278; G3HSI8 | *Gna11* |
| Q3HR10; P21279; G3IAG1 | *Gnaq* |
| Q80Z93; P23242 | *Gja1* |
| P26883 | *Fkbp1a; Fkbp1b; Fkbp5; H671_1g3255; I79_003335* |
| P29341; G3I8S7 | *H671_3g11210; H671_3g8899; Pabpc1; Pabpc4; Pabpc5* |
| P35441; CON__Q28194 | *Thbs1* |
| P47791; G3H609 | *Gsr* |
| P47962; G3HNJ6; G3HVH8 | *I79_006308; I79_008911; I79_011482; Rpl5* |
| P53026 | *Rpl10a* |
| P54227; A0A061IJR9; A0A061IHN3 | *H671_2g6450; Stmn1; Stmn3; Stmn4* |
| P56812; G3HF18 | *I79_009173; I79_016923; Pdcd5* |
| P57780 | *Actn4* |
| P60843; G3GY75 | *Eif4a1* |
| P61027; G3HC04; A0A061IPS8; G3GXQ2; Q9DD03 | *H671_1g1477; I79_002544; Rab13* |
| P61082; A0A061HTI3; G3I2Q4 | *I79_017697; Ube2m* |
| P61161 | *Actr2; I79_023522* |
| P61982; A0A061I2D3; G3IPD7; G3HA25 | *Ywhag* |
| P62259 | *Ywhae* |
| P62264; G3HS40 | *I79_013666; Rps14* |
| P62301; G3HMG1; A0A061IBD5 | *Rps13* |
| P62827; G3IHE5; G3HP89; G3IKJ4; A0A061I3A7 | *1700009N14Rik; H671_4g12852; I79_002522; Ran* |
| P62880; G3I668; A0A061I3S9 | *Gnb2* |
| P62889; G3H3C0; G3I078 | *I79_014424; I79_025029; Rpl30* |
| P62908; A0A061INH5 | *H671_1g2607; Rps3* |
| P62960 | *Ybx1* |
| P62962 | *Pfn1* |
| P63001; G3GVB5; A0A061I5F7; A0A061HWM0; A0A061I457; P60764 | *H671_4g11901; H671_7g18357; I79_022271; Rac1; Rac3* |
| P63028; G3H832 | *Tpt1* |
| Q76G10; P63037; G3I8V8; G3I8V9; G3ICK8 | *Dnaja1; I79_019990; I79_021406* |
| P63085; A0A061I5V3; A0A061I873; A0A061I951 | *Mapk1* |

| | |
|---|---|
| P63094; Q6R0H7; G3GWT7 | *Gnal; Gnao1; Gnas; Gnat2; Gnat3; H671_3g8733; I79_012092* |
| P63242; A0A061IN17; G3I948; Q8BGY2 | *Eif5a; Eif5a2; H671_1g1457* |
| P68372; Q60455; G3IG44; G3HCL2; Q9D6F9 | *H671_3g8577; Tubb4a; Tubb4b; Tubb6* |
| P68510; G3HK90 | *Ywhah* |
| P70349 | *Hint1* |
| Q06BU8; G3I255; P06151 | *H671_2g6037; H671_3g10390; I79_003584; I79_009741; I79_012728; I79_017480; Ldha; Ldhb; Ldhc* |
| Q3HR08; G3H860; P27601 | *Gna13* |
| Q3TXS7; G3ILH7 | *Psmd1* |
| Q60668; G3I782 | *Hnrnpd; Rbm31y* |
| Q61206; G3I0Q4 | *Pafah1b2* |
| Q61990; G3HV39 | *I79_008518; Pcbp2; Pcbp3* |
| Q64727; G3GWQ1 | *Vcl* |
| Q6ZWN5; A0A061HUC2; G3I351 | *H671_21637; Rps9* |
| Q76MZ3; G3I2B6; A0A061HU79 | *Ppp2r1a* |
| Q8BH43; G3I8T3 | *Wasf2* |
| Q8BH64; A0A061HXM3 | *Ehd2* |
| Q8BVE3; G3HSY7 | *Atp6v1h* |
| Q8MHC5; Q8MHC1 | *H2-D1; H2-T23; H671_1g3319; H671_1g4073; I79_013169; I79_013171; I79_016479; I79_022542* |
| Q8MHC2 | *H2-D1; I79_013138; I79_013172* |
| Q8MHC4 | *H2-D1* |
| Q8R081; A0A061HW75 | *Hnrnpl; I79_010758* |
| Q921M7 | *Fam49b; I79_025915* |
| Q99L47; G3I539 | *St13* |
| Q9CZY3; A0A061HZC1; G3HET8 | *Gm20431; I79_022620; I79_025517; Ube2v1* |
| Q9D2M8 | *I79_024907; Ube2v2* |
| Q9D358; G3HBH4 | *Acp1; I79_022830* |
| Q9D7S9 | *Chmp5* |
| Q9D8E6 | *I79_006744; Rpl4* |
| Q9DB05 | *H671_21071; Napa* |
| Q9EPP7 | *Ctsz* |
| Q9EQH3; A0A061IC10; G3I8H3 | *Vps35* |
| Q9JMG1; G3IHB4 | *Edf1* |
| Q9JMH6; G3HQL6 | *Txnrd1* |
| Q9QYB1; G3HMU4 | *Clic4* |
| Q9R119 | *H671_21681; I79_016276; Plaur* |
| Q9Z2U0; G3GWR8 | *Psma7* |
| V5QSN9; P46664; G3H7M3 | *Adss* |

Table S2: Detected proteins in the CHO-K1-derived exosomal fraction that are found significantly enriched vs soluble host cell protein (HCP), microvesicles (MV) or whole cell lysate (WCL).

| Protein name | Description | Enriched vs WCL | Enriched vs HCP | Enriched vs MV | Majority protein IDs |
|---|---|---|---|---|---|
| **6-phosphogluconate dehydrogenase, decarboxylating** | Pentose phosphate pathway protein | n.s. | ** | n.s. | G3IHY5 |
| **Actin-related protein 2** | ATP and actin filament binding | ** | n.s. | n.s. | Q5SW83; Q8BMA4 |
| **Alanine-tRNA ligase, cytoplasmic** | Protein biosynthesis | n.s. | ** | n.s. | G3IG23; Q3UD67; Q3TZ32; Q6XMP4; Q8R346; Q6PDM7 |
| **Alpha-mannosidase** | Carbohydrate binding and hydrolysis | **** | n.s. | n.s. | G3H559 |
| **ATP-citrate synthase** | Lipid metabolism | n.s. | n.s. | ** | Q3TED3; Q3V117; Q3UEA1 |
| **Basement membrane-specific heparan sulfate proteoglycan core protein** | Cell surface proteoglycan | ******** | ** | n.s. | G3HIM1 |
| **Beta-2-microglobulin** | Part of MHC-I complex | ** | n.s. | n.s. | G3H0S7 |
| **C-1-tetrahydrofolate synthase** | Folic acid metabolism | n.s. | ** | n.s. | G3H4Z5; A0A061HZ51 |
| **Cathepsin B** | Collagen and proteoglycan binding endopeptidase | ** | n.s. | n.s. | G3H0L9 |
| **Cathepsin L1** | Lysosomal protease | ** | n.s. | n.s. | G3INC5 |
| **Cathepsin Z** | Lysosomal peptidase | *** | n.s. | n.s. | Q9EPP7 |
| **Cell division control protein 42** | Membrane associated GTPase, involved in many signaling pathways | n.s. | ** | n.s. | G3HIM4; Q3UL78 |
| **Chloride intracellular channel protein** | Chloride ion channel | n.s. | ** | n.s. | Q543N5; G3HMU4 |
| **Chondroitin sulfate proteoglycan 4** | Transmembrane RTK signaling transducer | *** | n.s. | n.s. | G3H0E4 |
| **Clathrin heavy chain** | Cargo recognition, vesicle | n.s. | ****** | n.s. | Q5SXR6; Q80U89; A0A061I1I7; G3HJ89; Q3UPJ3 |

| | | | | | |
|---|---|---|---|---|---|
| | formation and endocytosis | | | | |
| **Clathrin light chain A** | Cargo recognition, vesicle formation and endocytosis | *** | n.s. | n.s. | G3IE25; B1AWE0; Q6PFA2; Q3THU7; A0A061IGC4; B1AWE1; B1AWD8; B1AWD9 |
| **Clusterin** | Extracellular chaperone | ******** | n.s. | ****** | G3HNJ3 |
| **Collagen alpha-1(III) chain-like isoform 2** | Extracellular matrix protein | n.s. | n.s. | *** | A0A061IIR5 |
| **Collagen alpha-1(XII) chain** | Extracellular matrix protein | n.s. | *** | ** | A0A061IAR2; A0A061I412; A0A061I8K0; A0A061I7N8; A0A061I5B5; G3HH34 |
| **Collagen alpha-2(V) chain** | Extracellular matrix protein | n.s. | **** | ** | A0A061IG65 |
| **Collagen, type V, alpha 1** | Extracellular matrix protein | n.s. | * | n.s. | B1AWB9 |
| **Cullin-associated NEDD8-dissociated protein 1** | Assembly factor of ubiquitin ligase complexes | * | n.s. | n.s. | G3GY17 |
| **EGF-containing fibulin-like extracellular matrix protein 1** | EGF signaling | n.s. | n.s. | **** | G3I9L7 |
| **EMILIN-1** | Extracellular matrix protein, cell migration | n.s. | ** | * | G3GXS2 |
| **Fatty acid synthase** | Lipid metabolism | n.s. | n.s. | **** | G3GXD7; A0A061I1B1; A0A061I3F6; A0A061HWV7 |
| **Ferritin** | Iron homeostasis | ***** | **** | ***** | G3IEF1 |
| **Fibronectin** | Cell surface binding | n.s. | **** | n.s. | G3I1V3 |
| **Filamin-B** | Actin binding, transmembrane protein anchor | n.s. | ** | n.s. | G3HFM4; A0A061IP39 |
| **Galectin** | Carbohydrate binding | n.s. | ** | n.s. | G3H7B3 |
| **Gamma-interferon-inducible lysosomal thiol reductase-like protein** | Lysosomal membrane protein | ** | n.s. | n.s. | A0A061IIU8; G3H4A9 |

| | | | | | |
|---|---|---|---|---|---|
| **Glypican-1** | Membrane bound proteoglycan, bearing heparan sulfate | ***** | n.s. | n.s. | G3H4T5; Q3U379 |
| **High mobility group protein B2** | Nuclear and secreted nucleic acid binding protein | * | n.s. | *** | G3HKY0 |
| **Histone H1** | DNA-binding protein | n.s. | ** | n.s. | G3HDU1; Q5SZA3 |
| **Histone H3** | DNA-binding protein | n.s. | **** | n.s. | G3H2T7; B9EI85; G3HPV9; G3HDS5; A1L0V4; A1L0U3; G3HDU8; G3HDT2; A0A1W2P768 |
| **Inosine-5-monophosphate dehydrogenase** | Involved in growth regulation, nucleotide synthesis and RNA/DNA metabolism | n.s. | ** | n.s. | A0A061I4E3; Q3UAT9; Q3U9N8; Q3UPJ2; A0A0A6YY72 |
| **Inter-alpha-trypsin inhibitor heavy chain H5** | Protease inhibitor | n.s. | *** | n.s. | G3GR64 |
| **Intercellular adhesion molecule 1** | Integrin binding | n.s. | ** | n.s. | Q9ERF7 |
| **Laminin subunit alpha-5** | Integrin binding | ****** | n.s. | ****** | G3HGW6 |
| **Laminin subunit beta-1** | Integrin binding | ** | * | **** | A0A061I094; G3I278; A0A061I6V4; Q3UHL7; E9QN70; B9EKB0 |
| **Laminin subunit gamma-1** | Integrin binding | ** | n.s. | ** | G3HG25 |
| **Legumain** | Involved in lysosomal degradation and EGFR recycling | *** | n.s. | n.s. | G3I1H5 |

| | | | | | |
|---|---|---|---|---|---|
| **Lipoprotein lipase** | Triglyceride metabolism | *** | n.s. | *** | A0A061IKA1; Q3UB54; Q8C562; Q4FJQ8; Q3UCD4; Q3UCH4; Q3UB98; Q3UC44; Q3U841; Q3U5U0; Q3UC81; Q3UAX2; G3H6V7; Q3U784; Q3UBE9; Q3UCZ2; Q3U6S8; Q3U715; Q8R4V8 |
| **Lysosomal alpha-glucosidase** | Carbohydrate binding and hydrolysis | ** | n.s. | n.s. | G3HTE5 |
| **Lysyl oxidase-like 1** | Histone lysyl deamination, cancer progression | n.s. | ** | n.s. | G3HEI6 |
| **Major vault protein** | Signaling scaffold protein, reported involvement in exosomal miRNA sorting | ****** | n.s. | n.s. | G3I4I9; A0A061IB55; Q8C2S9; Q3THX5; E9Q3X0 |
| **MKIAA1027 protein** | Cell adhesion and plasma membrane anchoring | n.s. | ** | n.s. | Q80TM2; Q3UHS6; Q0V930; Q3TBC3 |
| **Myosin-9** | Focal contact formation | n.s. | **** | n.s. | A0A061IEH1; A0A061IJ24; A0A061IL44; A0A061IFH1; G3IH63 |
| **Nidogen-1** | Sulfated membrane glycoprotein, cell matrix adhesion | ***** | n.s. | *** | G3I3U5 |
| **Nucleoplasmin domain-containing protein** | Histone and RNA binding (GO) | n.s. | ** | n.s. | Q3TAH3 |
| **Peptidyl-prolyl cis-trans isomerase** | Protein folding | n.s. | n.s. | *** | G3H533; Q9DCY1 |
| **Peroxidasin-like** | Extracellular matrix formation | ******* * | *** | ***** | G3HBI1; Q80U60; B2RX13; A0A1W2P6L9 |
| **Poly(rC)-binding protein 1** | RNA-binding protein | n.s. | ** | n.s. | G3HXL1 |
| **Poly(rC)-binding protein 2** | RNA-binding protein | n.s. | ** | n.s. | B2M1R7; Q3TF69; Q3TT81; G3HV39 |
| **Procollagen C-endopeptidase enhancer 1** | Collagen and heparin binding protein | ******* * | n.s. | ***** | A0A061I523; A0A061I8A2; |

| | | | | | |
|---|---|---|---|---|---|
| | | | | | G3I664; Q3UIP2; Q3TX39 |
| **Programmed cell death 6-interacting protein/ALIX** | MVB biogenesis and exosomal protein sorting | *** | n.s. | n.s. | G3H2N6; Q80Y09; Q6ZPS9 |
| **Prostaglandin F2 receptor negative regulator** | Lipid droplet organization, Reported association to CD9 and CD81 in myoblasts | ******* | **** | n.s. | G3H902; Q69ZJ6 |
| **Proteasome subunit beta** | Degradation of ubiquitinated proteins | **** | ** | n.s. | G3H303 |
| **Protein disulfide-isomerase A6** | Redox homeostasis | n.s. | ** | n.s. | Q3TML0; Q3TJL8; Q3THH1; Q9DAB4 |
| **SERPINH1** | Collagen binding | n.s. | ** | ** | G3IDD4; Q9Z1W7; Q3TWG9; Q8BVU9; Q8BV87; Q3TMD2; Q3TJK3 |
| **Sulfated glycoprotein 1** | Lysosomal glycoprotein | ***** | ** | n.s. | G3I1Y9 |
| **T-complex protein 1 subunit beta** | ATP and RNA binding chaperone | n.s. | ** | n.s. | G3HZ42; Q542X7; Q9JJD8 |
| **T-complex protein 1 subunit epsilon** | ATP and RNA binding chaperone | ** | *** | n.s. | G3H7K5 |
| **T-complex protein 1 subunit eta** | ATP and RNA binding chaperone | * | n.s. | n.s. | G3HYB7 |
| **T-complex protein 1 subunit gamma** | ATP and RNA binding chaperone | ** | *** | *** | G3HG83; Q3U4U6; E9Q133; Q3U0I3; Q3UJP4 |
| **T-complex protein 1 subunit zeta** | ATP and RNA binding chaperone | ** | n.s. | n.s. | G3I2M1; Q52KG9; Q3TIX8; Q3TI05; Q3TW97; Q3TI62 |
| **Tetraspanin** | MVB biogenesis and integrin binding | ** | n.s. | n.s. | G3HXZ3 |
| **Transitional endoplasmic reticulum ATPase** | MVB biogenesis, many other membrane formation processes | n.s. | ** | ** | Q8BNF8; G3HN14 |
| **Tripeptidyl-peptidase 1** | Lysosomal serine protease | **** | n.s. | ** | G3IDE4 |
| **Tubulointerstitial nephritis antigen-like** | Extracellular peptidase C1 family protein | ***** | *** | n.s. | G3H1W4 |

| | | | | | |
|---|---|---|---|---|---|
| **Ubiquitin activating enzyme E1** | Ubiquitin conjugation | n.s. | ** | n.s. | G3IBG3; B9EHN0; A0A1S6GWH5 |
| **Vacuolar protein sorting-associated protein VTA1-like** | MVB biogenesis | n.s. | n.s. | ** | G3HAV4; F8WJC2; Q3V4B3 |

Table S3: Detected proteins in the CHO-K1-derived exosomal fraction that are either significantly enriched in high viability samples (> 95 %) or in samples from the death phase (~80 – 60 % viability).

| Protein name | Description | FC: High viability/death phase | Significance | Majority protein IDs |
|---|---|---|---|---|
| **Matrix metalloproteinase-9** | Extracellular membrane proteinase | 3,45 | *** | G3H8V1 |
| **Lactadherin** | Phosphatidylserine binding and cellular uptake | 3,43 | * | G3ICD3 |
| **A disintegrin and metalloproteinase with thrombospondin motifs 1** | Extracellular membrane proteinase | 3,42 | *** | G3I3J3 |
| **Basigin** | Glycan receptor, cadherin binding and adhesion | 3,27 | ** | G3HF38; A0A061HZS2; A0A061HYF8 |
| **Laminin subunit beta-1** | Integrin binding | 3,22 | **** | A0A061I094; G3I278; A0A061I6V4; Q3UHL7; E9QN70; B9EKB0 |
| **Laminin subunit gamma-1** | Integrin binding | 3,13 | ** | G3HG25 |
| **Sodium/potassium-transporting ATPase subunit alpha** | Electrochemical gradient generation | 2,96 | * | A0A061IKK9; A0A061ILJ8; Q3TXF9; G3H8Z9; Q3UCH8; Q8R3M4 |
| **Integrin beta** | Signaling and adhesion | 2,96 | * | G3HZ55 |
| **Ras-related protein Rap-1b** | Signaling and regulation of exocytosis | 2,81 | * | G3GY22; Q52L50 |
| **Complement C1q tumor necrosis factor-related protein 1** | Cell signaling and collagen binding | 2,66 | * | G3H3E2 |
| **Glypican** | Regulation of signal transduction | 2,32 | ** | G3H4T5; Q3U379 |
| **Collagen alpha-1(V) chain** | Extracellular matrix protein | 2,28 | * | B1AWB9 |
| **Collagen alpha-1(III) chain-like isoform 2** | Extracellular matrix protein | 2,27 | * | A0A061IIR5 |
| **Lactadherin** | Phosphatidylserine binding and cellular uptake | 2,24 | * | G3IK05 |

| | | | | |
|---|---|---|---|---|
| **Collagen type IV alpha5 chain** | Extracellular matrix protein | 2,24 | * | P70165; Q61436; Q3TP61; G3HYQ7; Q80V57; Q8BNS7; F7CK55; Q9ESQ2; Q63ZW6; A0A1B0GRC0; A0A1B0GSI7; G3I7K9 |
| **Laminin subunit alpha-5** | Integrin binding | 2,23 | **** | G3HGW6 |
| **Collagen alpha-1(XII) chain** | Extracellular matrix protein | 2,20 | * | A0A061IAR2; A0A061I412; A0A061I8K0; A0A061I7N8; A0A061I5B5; G3HH34 |
| **Agrin** | Laminin binding | 2,05 | *** | G3I437; A0A061IHM8; A0A061IF33 |
| **Tubulointerstitial nephritis antigen-like** | Laminin binding peptidase | 1,87 | ** | G3H1W4 |
| **Complement C1r-A subcomponent** | Calcium ion binding | 1,81 | ** | G3GUR1 |
| **Lysyl oxidase-like 1** | Extracellular matrix crosslinking | 1,62 | * | G3HEI6 |
| **Polypeptide N-acetylgalactosaminyltransferase** | O-Glykosylation protein | 1,58 | * | A0A061ICG7; G3GX17; Q3UA32; Q3UM52 |
| **Heat shock protein Hsp20 containing protein** | Protein folding, stress response | 1,55 | * | A0A061I4R8; G3HA24 |
| **Histone H2A** | DNA-binding protein | 1,49 | ** | G3H154; Q8CA90 |
| **Neuroblast differentiation-associated protein AHNAK-like protein** | No information | 1,41 | ** | A0A061ID55 |
| **Metalloproteinase inhibitor 2** | Metalloprotease inhibitor | 1,31 | ** | G3H3E6; Q8BSJ3; Q6PI17; B1AQJ3 |
| **Basement membrane-specific heparan sulfate proteoglycan core protein** | Cell surface proteoglycan | 1,26 | * | G3HIM1 |

| | | | | |
|---|---|---|---|---|
| **Myosin-XVIIIa** | Cytoskeleton organization and NK cell activation | 1,05 | *** | G3I7W5; E9QA74; E9Q405; A0A1C7ZN10; K3W4L0; B2RRE2; E9QAX2 |
| **40S ribosomal protein S3a** | Ribosomal component | -1,09 | * | G3HKG8; Q564F3; Q3UAC2; Q9D1S3; Q3UJU5; Q3U5P8 |
| **Transitional endoplasmic reticulum ATPase** | ER formation | -1,21 | ** | Q8BNF8; G3HN14 |
| **Malate dehydrogenase** | TCA cycle and gluconeogenesis | -1,21 | ** | G3HDQ2 |
| **Transketolase** | Pentose phosphate pathway | -1,35 | ** | G3GUU5 |
| **Cofilin-1** | Actin binding | -1,36 | * | G3IDM2; Q544Y7; F8WGL3; Q9CX22 |
| **T-complex protein 1 subunit gamma** | ATP and RNA binding chaperone | -1,36 | **** | G3HG83; Q3U4U6; E9Q133; Q3U0I3; Q3UJP4 |
| **Septin-type G domain-containing protein** | GTP binding protein | -1,56 | * | Q8C2A3; Q3UNN1; E9Q9F5; E9Q1G8; A0A061I6G9; G3HTJ2; A0A061I443; Q5DTS3 |
| **Laminin receptor** | Ribosomal component | -1,66 | * | B2CY77; G3HQX0; A0A1L1SRW0 |
| **Proliferation-associated protein 2G4** | Ribosome-/RNA-binding protein, regulation of growth and apoptosis | -1,75 | ** | G3HRK0; Q05BN2; Q3UMW2; Q3TGU7 |
| **Protein S100-A10** | ANXA2 dimerization, vesicle budding and uptake regulation | -1,76 | * | G3HUU7; A0A061ILN9; A0A061IKI0; Q3UF30; Q3TC45 |

| | | | | |
|---|---|---|---|---|
| **Elongation factor 1-delta** | Ribosome-associated translation regulator | -1,80 | * | G3I5H3; A0A061IEA0; A0A061IGU4; A0A061ID94; A0A061IJX7; A0A061IE95; A0A061IHT6 |
| **Fatty acid synthase** | Lipid metabolism | -1,80 | *** | G3GXD7; A0A061I1B1; A0A061I3F6; A0A061HWV7 |
| **Alanine--tRNA ligase, cytoplasmic** | Protein biosynthesis | -1,80 | ** | G3IG23; Q3UD67; Q3TZ32; Q6XMP4; Q8R346; Q6PDM7 |
| **Alpha-enolase** | Glycolysis | -1,83 | ** | G3IAQ0 |
| **Heat shock protein 90 alpha** | Chaperone, cellular stress response | -1,84 | ** | Q71LX8; G3HC84 |
| **Filamin-B** | Actin binding | -1,86 | ** | G3HFM4; A0A061IP39 |
| **Endoplasmic reticulum chaperone BiP** | ER chaperone | -1,91 | *** | G3I8R9; Q3U9G2; Q3TWF2; Q3TKF8; Q3TI47; Q9DC41; Q3U7T8; Q3U6V3; Q3UEM8 |
| **T-complex protein 1 subunit zeta** | ATP and RNA binding chaperone | -1,95 | * | G3I2M1; Q52KG9; Q3TIX8; Q3TI05; Q3TW97; Q3TI62 |
| **Lipase** | Triglyceride metabolism | -1,96 | * | G3HQY6 |
| **NSFL1 cofactor p47** | Intermediate filament cytoskeleton | -1,99 | * | G3H6Y6; Q3UVN5; Q3KQQ1; A2AT02 |
| **Stress-induced-phosphoprotein 1** | HSP90 binding, stress response | -2,03 | ** | G3I877; Q3THQ5 |
| **Heterogeneous nuclear ribonucleoprotein A2/B1** | mRNA binding and transport | -2,03 | * | B7ZP22; G3H2J8; A0A0N4SUM2 |

| | | | | |
|---|---|---|---|---|
| **Heterogeneous nuclear ribonucleoprotein D0** | mRNA binding and transport | -2,05 | ** | F6ZV59; G3I782; G5E8G0; G3X9W0; E9Q5B6; F6SHF3 |
| **Clathrin light chain** | Cargo recognition, vesicle formation and endocytosis | -2,07 | * | Q3TWZ9; Q3TJ95; A0A061IGR6; G3H194; A0A061IEM0; A2VCP8; F7BHJ0 |
| **Proteasome subunit alpha type** | Protein degradation | -2,08 | * | Q542H2; Q3UIT9; Q3TN31; G3GWR8; Q3THL2 |
| **Heterogeneous nuclear ribonucleoprotein** | mRNA binding and transport | -2,11 | ** | Q5FWJ5; Q3TL71; Q3TUA1; B2M1R6; Q3TJ38; Q3U6X2; Q3U9Q3; Q3TG17; H3BLL4; G3IAS8; H3BKD0; H3BK96; A0A286YDM3 |
| **Aldehyde dehydrogenase family 16 member A1** | Acetaldehyde detoxification | -2,11 | * | G3I000 |
| **Proteasome subunit alpha type** | Protein degradation | -2,13 | * | A0A061I2E1; A0A061I019; E0CXB1 |
| **T-complex protein 1 subunit delta** | ATP and RNA binding chaperone | -2,13 | *** | Q564F4; Q3UJZ8; Q3TII0; G5E839 |
| **T-complex protein 1 subunit theta** | ATP and RNA binding chaperone | -2,23 | ** | Q3UL22; Q3UKQ2; Q6A0F1; Q9WVS5; Q8BVY8; Q9CS06; H3BL49 |

| | | | | |
|---|---|---|---|---|
| **Serpin H1** | Chaperone, collagen binding | -2,26 | * | G3IDD4; Q9Z1W7; Q3TWG9; Q8BVU9; Q8BV87; Q3TMD2; Q3TJK3 |
| **Cadhepsin Z** | Lysosomal protease | -2,32 | * | Q9EPP7 |
| **T-complex protein 1 subunit beta** | ATP and RNA binding chaperone | -2,37 | **** | G3HZ42; Q542X7; Q9JJD8 |
| **UTP-glucose-1-phosphate uridylyltransferase** | Glucose/Glycogen metabolism | -2,38 | ** | G3HDQ1; Q8R0M2; Q3U548 |
| **60S ribosomal protein L22** | Ribosomal component | -2,43 | * | Q4VAG4 |
| **Pre-mRNA-processing factor 19** | mRNA splicing | -2,44 | *** | G3IE04 |
| **Protein DJ-1** | Involved in a multitude of regulation processes | -2,53 | ** | G3IEU2 |
| **Alpha-centractin** | Cytoskeleton | -2,57 | * | G3HXW9 |
| **T-complex protein 1 subunit alpha** | ATP and RNA binding chaperone | -2,58 | *** | G3HGP6 |
| **Farnesyl pyrophosphate synthetase** | Isoprenoid biosynthesis | -2,65 | ** | G3HC39 |
| **Protein disulfide-isomerase A6** | Cellular redox homeostasis | -2,68 | * | G3HB04 |
| **Septin-11** | Cytoskeletal GTPase | -2,73 | ** | G3IC99; A0A0J9YTY0; A0A0J9YUL3 |
| **40S ribosomal protein S12** | Ribosomal component | -2,83 | ** | Q6ZWZ6; A0A061IHI0; F7AEH4; G3I737; A0A1W2P7A1 |
| **60S ribosomal protein L13** | Ribosomal component | -2,85 | * | A0A061IET8; G3GRR8 |
| **Heat shock protein 90 beta** | Chaperone, cellular stress response | -2,86 | ** | G3HQM6; Q91V38; Q3UAD6; Q3UBU0; Q3TUD6; Q8CCY5; F7C312 |

| | | | | |
|---|---|---|---|---|
| **Plectin** | Intermediate filament cytoskeleton | -2,96 | ** | Q6S385; Q6S387; Q6S388; Q6S392; Q6S393; E9Q3W4; Q6S390 |
| **T-complex protein 1 subunit theta** | ATP and RNA binding chaperone | -3,01 | ** | G3GT06; A0A061IBK9 |
| **Proteasome subunit alpha type** | Protein degradation | -3,09 | *** | Q9DCD8; Q58EV4; G3I9G7; Q3TEL1; E0CX62 |
| **Lamin-A/C** | Nuclear lamina component | -3,12 | ** | G3HG95 |
| **Peroxiredoxin-1** | Cellular redox homeostasis | -3,38 | *** | G3GYP9 |
| **L-lactate dehydrogenase** | Lactate metabolization | -3,59 | ** | Q06BU8; G3I255 |
| **Protein disulfide-isomerase** | Cellular redox homeostasis, cysteine bond rearrangement | -4,20 | ** | A0A061HWS2; G3IDT6 |

Table S4: Detected micro RNA (miRNA) species in the CHO-K1-derived exosomal (EXO) fraction and the respective growth phase they can be found in. Additionally, it is indicated whether the respective miRNA is either significantly down or upregulated in EXO compared to whole cell lysate (WCL).

| miRNA | Found in log. phase | Found in stat. phase | Found in death phase (80 % via.) | Found in death phase (60 % via.) | Enrichment in EXO vs WCL |
|---|---|---|---|---|---|
| cgr-let-7b | + | + | + | + | n.s. |
| cgr-let-7c-1 | + | + | + | + | Down (*) |
| cgr-let-7f | + | + | + | + | Down (******) |
| cgr-mir-10a | + | + | + | + | n.s. |
| cgr-mir-10b | + | + | + | + | n.s. |
| cgr-mir-146b | + | + | + | + | Down (****) |
| cgr-mir-191 | + | + | + | + | Up (*) |
| cgr-mir-21 | + | + | + | + | Down (******) |
| cgr-mir-22 | + | + | + | + | Up (***) |
| cgr-mir-221 | + | + | + | + | n.s. |
| cgr-mir-25 | + | + | + | + | Up (*****) |
| cgr-mir-26a-2 | + | + | + | + | Down (***) |
| cgr-mir-30c-1 | + | + | + | + | n.s. |
| cgr-mir-30d | + | + | + | + | Down (**) |
| cgr-mir-378 | + | + | + | + | Up (***) |
| cgr-mir-423 | + | + | + | + | Up (************) |
| cgr-mir-92a | + | + | + | + | Up (*****) |
| cgr-let-7a-2 | + | + | + | + | Down (***) |
| cgr-mir-181a | + | + | + | + | n.s. |
| cgr-let-7c-2 | | + | + | + | Down (*) |
| cgr-let-7d | | + | + | + | n.s. |
| cgr-let-7e | | + | + | | n.s. |
| cgr-let-7g | | + | + | + | Down (**********) |
| cgr-let-7i | | + | + | + | Down (*******) |
| cgr-mir-100 | | + | + | + | n.s. |
| cgr-mir-125a | | + | + | + | Down (*****) |
| cgr-mir-128 | | + | + | + | n.s. |
| cgr-mir-130a | | + | + | + | Up (******) |
| cgr-mir-132 | | + | + | + | n.s. |
| cgr-mir-140 | | + | + | + | Up (*******) |
| cgr-mir-151 | | + | + | + | n.s. |
| cgr-mir-16 | | + | + | + | Down (***) |
| cgr-mir-16-2 | | + | + | + | Down (***) |
| cgr-mir-181b | | + | + | + | Up (**) |
| cgr-mir-181c | | + | + | + | Up (*) |
| cgr-mir-185 | | + | + | + | n.s. |
| cgr-mir-186 | | + | + | + | Down (*) |
| cgr-mir-196a | | + | + | + | Down (***) |
| cgr-mir-199a | | + | + | + | n.s. |
| cgr-mir-199a-2 | | + | + | + | n.s. |
| cgr-mir-20a | | + | + | + | Down (****) |
| cgr-mir-222 | | + | + | + | n.s. |

| | | | | | |
|---|---|---|---|---|---|
| **cgr-mir-23a** | | + | + | + | n.s. |
| **cgr-mir-24** | | + | + | + | Up (*****) |
| **cgr-mir-24-1** | | + | + | + | Up (****) |
| **cgr-mir-26a-1** | | + | + | + | Down (***) |
| **cgr-mir-26b** | | + | + | | Down (****) |
| **cgr-mir-27a** | | + | + | + | n.s. |
| **cgr-mir-27b** | | + | + | + | n.s. |
| **cgr-mir-28** | | + | + | + | Up (*****) |
| **cgr-mir-298** | | + | | + | n.s. |
| **cgr-mir-3074-1** | | + | + | + | Up (****) |
| **cgr-mir-3074-2** | | + | + | + | Up (***) |
| **cgr-mir-30b** | | + | + | + | n.s. |
| **cgr-mir-30c-2** | | + | + | + | Down (***) |
| **cgr-mir-30e** | | + | + | + | Down (******) |
| **cgr-mir-31** | | + | + | | Down (***) |
| **cgr-mir-320a** | | + | + | + | n.s. |
| **cgr-mir-322** | | + | + | + | Down (**) |
| **cgr-mir-34b** | | + | + | + | Up (*) |
| **cgr-mir-34c** | | + | + | + | n.s. |
| **cgr-mir-3535** | | + | + | + | Down (****) |
| **cgr-mir-425** | | + | + | + | n.s. |
| **cgr-mir-455** | | + | | | n.s. |
| **cgr-mir-486** | | + | + | + | n.s. |
| **cgr-mir-503** | | + | + | + | Down (*) |
| **cgr-mir-574** | | + | + | + | n.s. |
| **cgr-mir-615** | | + | + | + | n.s. |
| **cgr-mir-671** | | + | + | + | n.s. |
| **cgr-mir-7a** | | + | | | n.s. |
| **cgr-mir-872** | | + | + | + | n.s. |
| **cgr-mir-92b** | | + | + | + | n.s. |
| **cgr-mir-99a** | | + | + | + | n.s. |
| **cgr-mir-106b** | | + | + | + | n.s. |
| **cgr-mir-125b-2** | | + | + | + | n.s. |
| **cgr-mir-146a** | | + | | + | n.s. |
| **cgr-mir-149** | | + | + | + | n.s. |
| **cgr-mir-181d** | | + | + | + | n.s. |
| **cgr-mir-192** | | + | + | + | n.s. |
| **cgr-mir-193b** | | + | + | | n.s. |
| **cgr-mir-19b** | | + | + | + | Down (******) |
| **cgr-mir-29a** | | + | + | + | Up |
| **cgr-mir-328** | | + | + | + | n.s. |
| **cgr-mir-330** | | + | | | n.s. |
| **cgr-mir-484** | | + | + | + | n.s. |
| **cgr-mir-542** | | + | + | + | Down (****) |
| **cgr-mir-652** | | + | + | + | n.s. |
| **cgr-mir-674** | | + | + | + | n.s. |
| **cgr-mir-93** | | + | + | + | n.s. |

| | | | | | |
|---|---|---|---|---|---|
| **cgr-mir-101a** | | | + | + | n.s. |
| **cgr-mir-101b** | | | + | + | n.s. |
| **cgr-mir-103** | | | + | + | n.s. |
| **cgr-mir-107** | | | + | + | n.s. |
| **cgr-mir-125b** | | | + | | n.s. |
| **cgr-mir-1260** | | | + | | n.s. |
| **cgr-mir-148b** | | | + | | n.s. |
| **cgr-mir-155** | | | + | | n.s. |
| **cgr-mir-15a** | | | + | + | n.s. |
| **cgr-mir-17** | | | + | | n.s. |
| **cgr-mir-1839** | | | + | + | n.s. |
| **cgr-mir-1843** | | | + | | n.s. |
| **cgr-mir-19a** | | | + | | n.s. |
| **cgr-mir-210** | | | + | + | n.s. |
| **cgr-mir-301a** | | | + | + | Down (*) |
| **cgr-mir-3102** | | | + | | n.s. |
| **cgr-mir-340** | | | + | + | n.s. |
| **cgr-mir-342** | | | + | + | n.s. |
| **cgr-mir-361** | | | + | | n.s. |
| **cgr-mir-365-2** | | | + | | n.s. |
| **cgr-mir-450a** | | | + | | n.s. |
| **cgr-mir-450b** | | | + | | n.s. |
| **cgr-mir-505** | | | + | | n.s. |
| **cgr-mir-664** | | | + | | n.s. |
| **cgr-mir-744** | | | + | + | n.s. |
| **cgr-mir-99b** | | | + | + | n.s. |
| **cgr-mir-29b** | | | | + | n.s. |
| **cgr-mir-344** | | | | + | n.s. |
| **cgr-mir-377** | | | | + | n.s. |
| **cgr-mir-126a** | | | | + | n.s. |
| **cgr-mir-497b** | | | | + | n.s. |
